# Reputation-based reciprocity in human–bot and human–human networks

**DOI:** 10.1093/pnasnexus/pgaf150

**Published:** 2025-05-09

**Authors:** Ashley Harrell, Margaret L Traeger

**Affiliations:** Department of Sociology, Duke University, Durham, NC 27705, USA; Department of Information Technology, Analytics, and Operations, University of Notre Dame, Notre Dame, IN 46556, USA

**Keywords:** cooperation, human–robot interaction, network science, indirect reciprocity, generosity

## Abstract

People help those with a reputation for helping others; as a result, they are more likely to behave generously when reputational concerns are present. Because people are increasingly making helping decisions in the presence of both humans and AI in “hybrid systems,” here we ask whether and how reputation-based reciprocity (RBR) promotes generosity in human–bot networks, compared with human-only ones. In two experiments—one where interactants were embedded in a patterned indirect reciprocity network and either knew or did not know that bots were present and another entailing one-shot interactions between humans and bots—we demonstrate that RBR is significantly less effective at fostering generosity in hybrid systems. At the network level, people are less generous when they know bots are present. In line with prior work, our findings suggest that this is driven by altered norms about helping in (known) hybrid networks governed by RBR: people do not believe bots deserve help like humans do, reducing overall generosity. In one-shot dyadic interactions, we likewise demonstrate that people are less willing to help bots even when they can receive reputational rewards for helping and even toward bots that have reputations for helping humans (or bots). People are also less likely to help people who help bots (compared with people who help people) and punish people who fail to help bots (compared with people who fail to help people). Adding bots to RBR networks affects not only humans' prosocial behavior, but also their evaluations of generosity toward human and bot alters.

**Significance Statement**

Prior work has demonstrated that reputations promote generosity: people engage in “reputational giving” (behaving generously when their behavior may be observed by a third party who can, in turn, help them) and “rewarding reputations” (helping those who have a reputation for helping others). Understanding whether and how the addition of machines into human social networks affects these reputation-based reciprocity (RBR) processes is critical, as humans and machines increasingly must help, and be helped by, each other. Our two experiments reveal that RBR fails to generate high levels of giving in human–bot networks, compared with human-only ones, and show how the addition of bots into networks governed by RBR can weaken norms of human generosity.

## Introduction

People are sensitive to reputational rewards and bestow benefits on those with reputations for generosity (1–6). The prospect of being helped as a result of a reputation for being generous, or of *not* being helped as a result of a reputation for selfishness, drives prosocial behavior, even among unrelated individuals. As a result, reputation-based reciprocity (RBR) (also commonly referred to as *downstream indirect reciprocity*)—where A helps B and then a third party C helps A—is a key explanation for the evolution of prosocial behavior (7–12).

RBR has thus far been shown to promote prosocial behavior in small and large networks of *human* interactants (4, 13–18). But people increasingly must work with bots and other forms of AI in what have been referred to as “hybrid systems” (19–21). This includes settings where the interactants—that is, both humans *and* bots—can help, and be helped by, each other, including collaborative tasks (19, 22), team decision-making (23, 24), moving about in physical space (25, 26), and mediating conflict (27, 28). Here, we ask: how are human helping decisions altered when bots are introduced into networks governed by RBR processes?

One stream of prior research has argued that people commonly perceive machines as social actors (29–31) and treat them akin to how they treat humans (32). From this perspective, RBR may operate similarly in hybrid networks as it does in human-only ones: decisions to behave generously will remain high when RBR is possible, regardless of the make-up of the network. Yet, other work has argued that there are clear differences in how people judge morally relevant behaviors enacted *by* machines versus humans and *toward* machines versus humans (33). This work argues that social norms about (not) helping bots are distinct from those about (not) helping humans (34–36), such that network-level norms about giving weaken when bots are introduced to the network (26).

For example, previous research demonstrates that people are less likely to engage in a variety of prosocial behaviors when they are interacting with a bot, compared with another person (or a bot they are told is a person) (36–39). They are also more willing to exploit benevolent AI than they are benevolent humans (40). Other related work suggests that people judge prosocial (and antisocial) behaviors toward other humans versus machines differently: observers view humans' aggressive driving behavior toward an autonomous vehicle to be more acceptable and less immoral compared with the same behavior directed toward another human-driven vehicle (41). Even young children view robots and humans as mentally and emotionally distinct (42), and they evaluate inequality as less unfair when the disadvantaged recipient is a robot, compared with a human (43).

Additionally, in a set of experiments particularly relevant to the work described here, Makovi et al. (36) examine a third-party punishment task where (potential) “helpers” and third-party “punishers” could receive benefits from a downstream “trustor.” They demonstrate that people are less willing to punish helpers who do not share their resources with bots, compared with those who do not share their resources with people. Still further, while trustors bestow trust on those who share their resources and on those who punish selfishness, these trust benefits are reduced when bots are embedded in the network. The differences are driven by people's uncertainty about helping norms in hybrid networks; providing people with information about the consensuality of helping norms can reduce the differences in trust gains in hybrid versus human-only collectives (36).

Because the prior work described above was designed to assess different questions, it has not directly compared generosity in settings where RBR is possible to settings where it is not. Nor has it embedded participants in networks where they can simultaneously help, and be helped by, a mix of participants and bots. Our work builds on these studies in several ways. First, theoretical and empirical models distinguish between two processes in RBR (13, 44). “Reputational giving” occurs when role-occupant A helps (or does not help) B in the presence of third party C. Then, role-occupant C reciprocates A's behavior by helping (or not helping) A (“rewarding reputations”). Either or both of these processes may be disrupted when humans interact in hybrid systems governed by RBR. In our first experiment, which we call the *chain reciprocity study*, we assess helping patterns in fixed networks involving the transfer of resources via RBR. Any given decision to help in this study involves simultaneously helping those based on their reputation and the prospect of being helped based on one's own reputation. In one condition of this study, participants are aware that they can help, and be helped by, either a bot or a participant, fully crossed. This allows us to establish if network-level helping patterns in RBR settings are less likely to facilitate generosity when bots are embedded in the network, and if this is driven by people who are positioned in the network to help bots (versus people), people who are positioned in the network such that they may be helped *by* bots (versus people), or both.

Second, prior work has not yet directly compared generosity patterns in networks where RBR is or is not possible, when bots are or are not present among interactants. In the second experiment we describe here, which we call the *one-shot giving study*, we use a series of one-shot decisions (13, 36, 45) to examine giving in private compared with giving when RBR is possible. This allows us to establish whether RBR promotes generosity when some of the interactants involved are bots and, if so, if it does to the same extent as in human-only interactions. We also more clearly distinguish between the reputational giving and rewarding reputations processes in the second experiment, evaluating whether and which *specific* processes in RBR are altered—reputational giving, rewarding reputations, or both—when some or all of the role-occupants are bots.

**Fig. 1.**
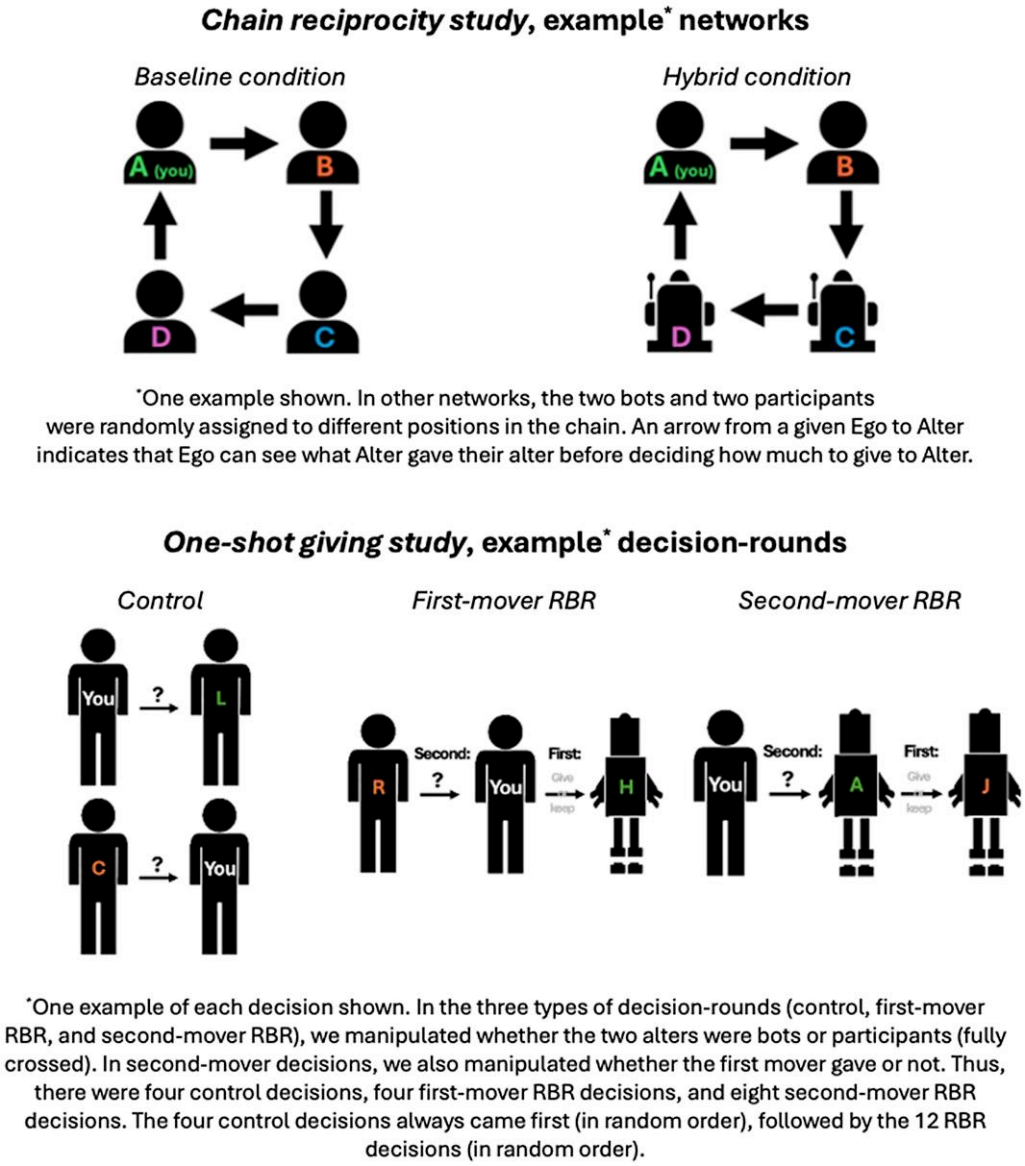
Outline and example conditions, *chain reciprocity study* (top) and *one-shot giving study* (bottom). *Top* Examples of networks in the *chain reciprocity study*, shown from the perspective of a participant randomly assigned to the A position. In the *hybrid* condition (right), participants were aware that they were paired with two bots and one other participant and knew the bots' randomly assigned locations in the network (e.g. they could see that a bot would make the decision to give to them). In the *baseline* condition (left), participants were also paired with one other participant and two bots, but they were not aware that bots were in the network and were always shown an image with four humans on the screen. *Bottom* Example conditions in the *one-shot giving study*.

Our *chain reciprocity study* involved fixed relations through which help could flow in a repeated setting. The goal of the chain reciprocity study was to test whether human helping behaviors are altered when known bots are introduced into networks governed by RBR. In this study, participants and bots were embedded in a closed four-agent network (13); an example is shown in the top row of Fig. 1. The agent occupying position A could give benefits (tokens, worth money, which increased in value when they were given) to B. Then, D learned what A gave to B before giving benefits to A; then, C learned what D gave to A before giving benefits to D; B learned what C gave to D before giving benefits to C, and, closing the “chain,” A learned what B gave to C before deciding how much to give to B in what began the second “round” of interaction. This cyclic exchange continued for several rounds (13). As a result, RBR was possible: any given agent (ego) knew how many tokens their alter had given to someone else (but no other information was provided, including what they themselves had received in the previous round, to avoid income effects that might complicate the mechanism we were testing (13, 46)) before deciding how many tokens to send to that alter. Likewise, ego's own helpful (or selfish) behavior was available to the agent assigned to give to ego, before they decided how much to benefit ego.

We randomly assigned two participants and two bots to a position in the network and randomly assigned each network to one of two conditions. In the *hybrid* condition, participants were told in advance that they would be paired with two bots and one other participant; an image on their screen while they were making token-giving decisions showed who in the network was a bot and who was a participant (Fig. 1). In the *baseline* condition, we told participants they were interacting with three other participants and the image shown on their screen suggested that everyone in the network was a human. However, they interacted with two bots and one other participant just as those in the hybrid condition did. The random assignment of humans and bots to positions within the chain network meant that our participants were sometimes in a position to help (by sending tokens to) a bot, be helped by (by receiving tokens from) a bot, or both. We control for these patterns in our analyses and assess how they affected participants' decisions. The deception in one condition of the chain reciprocity study (i.e. holding constant the bots versus humans make-up of the network—rather than, in the baseline condition, pairing four participants with each other) allowed us to test whether the *known* presence of bots in a hybrid network impacts the giving behaviors of our human participants, controlling for the actual presence of preprogrammed behaviors (see (47, 48) for more on deception and acceptable forms of deception in experimental research). Participants were fully debriefed after the study concluded, in line with best-practice recommendations and prior work showing that being deceived in one study does not affect the validity of others (49–52).

The bots in our study needed to be programmed. We chose a relatively simple strategy when deciding how to program them: bots were “strong reciprocators”—that is, they discriminately rewarded generosity with generosity, and punished selfishness with selfishness (53–55). In practice, this meant that our bots gave their alter the same number of tokens that they learned the alter had given. We chose strong reciprocation as the bots' strategy because prior work has shown that while agents programmed to engage in discriminant prosocial behavior promotes generosity, agents programmed to be indiscriminately generous or indiscriminately selfish *undermines* it (37, 56). These strategies would therefore have been a relatively weaker test of whether the presence of bots in networks governed by RBR hinders generosity. Having our bots engage in strong reciprocity is a more conservative test of whether bots reduce generosity in RBR networks, even when they engage in behavior that is known to cause prosociality.

At two points midway through the task, participants completed two sets of questions after seeing what their alter gave, but before making their own decision about giving to the alter. One was a scale measuring ego's concern for how they were viewed by the alter who was assigned to give to them (57–59). This set of items assessed whether participants were concerned with their reputation—as we would expect in a setting governed by RBR—and whether (and how) this differed when participants interacted with known bots. The second set of questions measured perceptions that the alter to whom they were assigned to give actually deserved help. We used these to assess whether endorsing giving toward one's alter (holding constant the alter's own giving decision) differed when participants interacted with known bots.

The *one-shot giving study* allows us to answer several questions that the chain reciprocity study does not, as well as address several limitations of the chain reciprocity study. First, the chain reciprocity study did not manipulate whether reputational concerns via the prospect of RBR were present or absent—they were always present. The one-shot giving study included control decisions where participants could help (or not help) an alter without concern for their reputation (i.e. in private). This allows us to better assess whether RBR increases giving in hybrid settings like it does in human-only ones, compared with settings where RBR is not possible.

Second, in the chain reciprocity study, ego's decision to help was simultaneously affected by both (i) whether, and how much, the alter had helped someone else (i.e. rewarding reputations) and (ii) the presence of the alter who could give to ego, after knowing what ego had done (i.e. reputational giving). The one-shot giving study allowed us to disentangle these two processes and understand how the presence of bots as role-occupants impacts each distinct process (because participants made a series of decisions in both scenarios separately).

Finally, the one-shot giving study did not use deception–participants were always informed accurately about whether they would be paired with a participant or a bot in a given round—and bots did not need to be programmed to follow a specific decision rule (e.g. strong reciprocity) over multiple rounds (as they did in the chain reciprocity study). Instead, in the one-shot giving study, we paired participants with real other participants or bots who had either given tokens or not. This allowed us to assess participants' behaviors toward bots in a setting more decoupled from multiround strategic decision-making.

In the one-shot giving study, participants were told that they would be making decisions with a mix of bots and humans in each of several rounds. However, they would never be paired with the same other(s) in any given round (as a result, as in the chain reciprocity study, direct reciprocity was not possible). Thus, this study, as in prior work (13, 44), assesses RBR in several forms in a series of one-shot decisions such that each person serves as their own control, allowing all participants to be exposed to instructions of the same length and complexity (rather than some participants reading simpler control decision instructions and others reading more complicated RBR decision instructions). All participants first completed a set of four *control* decisions (see the bottom panel of Fig. 1) in random order. In the control decisions, RBR was not possible: participants would make a decision to give (or not give) a one-token endowment to an alter, but their decision was private. They were also told that someone else would be assigned to decide whether to give a token to them at the same time, as shown in Fig. 1 (however, as in the chain reciprocity study, participants never learned whether or how much had been given to them). We manipulated whether the others in the scenario (i.e. the alter to whom ego could send benefits and the alter from whom ego could receive benefits) were bots or other participants, yielding four control decisions in total. These decisions always came first, as a measure of participants' baseline giving behavior before they were exposed to information about what others had given (as they were in the RBR decisions).

The remaining 12 decisions came after, in random order. In four of them, participants made “reputational giving” decisions (see Fig. 1)—they were told that they (ego, A) could give their token (or not) to an alter (B); then, a third party (C) would learn what ego had done before making the decision to give their token (or not) to ego. As in the control decisions, we manipulated whether the alters (B and C) were bots or real other participants, fully crossed. In the remaining decisions, participants could “reward reputations” (Fig. 1)—they could give their token to an alter (A) after learning whether A had helped someone else (B). Again, we manipulated whether A and B were bots or other participants. We also manipulated, in each participant and bot make-up, whether they were matched with an A who they were informed had decided to help B, or not, for eight decisions total.

## Results

Table S1 contains information about our sample demographics for both studies. In the *chain reciprocity study*, 2,192 participants (1,052 in the baseline condition and 1,140 in the hybrid condition; our preregistered target sample size was 2,000) made 23,995 decisions about token-giving. We used multilevel linear models to account for the dependencies in the nested data (multiple rounds were nested in participants, and participants were nested in networks with one real other participant). The outcome variable was the number of tokens the participant (ego) sent to the alter to whom they were able to give, which could range from 0 to 10.

We analyzed token-giving differences across conditions in a model that included terms for round, whether the alter to whom ego could *give* benefits was a bot, whether the alter from whom ego could *receive* benefits was a bot (whether the diagonal alter was a bot could be perfectly predicted from the terms for whether the other two alters were bots, because participants were always paired with exactly two bots in the network), and the information that ego received about what their alter had given in the round. Because of the inclusion of the latter term, decisions made in the first round by participants assigned to the A position were dropped, because they received no information about what their alter had given in the round, by necessity. As a result, the number of decision rounds actually included in the models was 23,416. The SI contains analyses showing that behaviors by the “first movers” were substantively the same as those described below (Table S3). It also displays additional models assessing the robustness of our results for both studies (Tables S4–S23), including models using cluster robust SEs instead of multilevel models as an alternative way of accounting for the nested data, models demonstrating that the results described below hold when the outcome is binarized (“gave all tokens” versus “did not give all tokens,” see also Fig. S3), and bootstrapped estimates.

Our initial model (model 1 in Table 1) contained just the main effects. This model confirmed that when participants were aware that they were interacting with bots (i.e. in the hybrid condition), they gave fewer tokens to their alter (holding constant whether the alter to whom they could give was a bot or not, which we turn to next), compared with when they were not aware (*B* = −0.54, *P* < 0.001). While participants did reciprocate generosity with generosity (the amount the alter gave significantly predicted how much ego gave the alter, *B* = 0.38, *P* < 0.001)—and giving behaviors stayed high over time (*B* = 0.02, *P* < 0.001, see also Fig. S2) (follow-up models confirmed that these patterns were similar across conditions: neither the *round* × *hybrid condition* interaction nor the *amount alter gave* × *hybrid condition* interaction was significant)—participants embedded in RBR networks with known bots transferred significantly fewer resources across the rounds of the task.

**Table 1.** Token-giving, chain reciprocity study.

	Model 1			Model 2		
Predictors	Est.	SE	95% CI	Est.	SE	95% CI
Intercept	4.85^b^	0.17	4.52–5.18	4.54^b^	0.23	4.10–4.99
Hybrid condition (H)	−0.54^b^	0.10	−0.73—−0.35	0.07	0.31	−0.54–0.67
The alter that ego can *give to* is a bot	−0.17	0.11	−0.39–0.05	0.21	0.16	−0.11–0.53
The alter that ego can *receive from* is a bot	−0.07	0.11	−0.29–0.15	−0.03	0.16	−0.35–0.30
Round	0.02^b^	0.00	0.01–0.02	0.02^b^	0.00	0.01–0.02
Amount Alter_give to_ gave their alter this round	0.38^b^	0.01	0.37–0.39	0.38^b^	0.01	0.37–0.39
H × Alter_give to_ is a bot				−0.73^a^	0.23	−1.17—−0.29
H × Alter_receive from_ is a bot				−0.09	0.23	−0.54–0.35
Observations	23,416			23,416		
Marginal *R*^2^/conditional *R*^2^	0.199/0.638			0.202/0.639		
AIC	101,017.035			101,011.223		

^a^ *P* < 0.01.

^b^ *P* < 0.001.

Multilevel linear models with random intercepts for the multiple decision rounds nested in participants (*n* = 2,192) nested in networks (*n* = 1,293) with one real other participant. Round ranges from 0 to 10. The first-round decisions for participants in the A position are dropped because they did not receive information about the amount their alter had given in the first round, by necessity. The outcome variable is the number of tokens ego gave their alter, ranging from 0 to 10.

At first glance, the actual location of bots in the network compared with their human counterparts did not appear to impact participants' giving decisions (Table 1, model 1, *B* = −0.17, *P* = 0.14 and *B* = −0.07, *P* = 0.53 for whether the alter that ego gave to and received from, respectively, was a bot). But note that only in about half of our observations were participants *aware* that the bot in that location was a bot. We therefore tested for interactions between the hybrid condition (where participants were aware that bots were, in fact, bots) and (i) whether the alter that ego could *give to* was a bot and (ii) whether the alter that ego could *receive from* was a bot.

The results from this model (model 2 in Table 1) suggested that the lower levels of giving in hybrid networks were driven by those participants who could help bots (*hybrid condition × alter that ego can give to is a bot* interaction, *B* = −0.73, *P* < 0.001). On the contrary, knowing that one would (potentially) receive benefits *from* a bot (versus from another participant) did not appear to impact giving behavior (*B* = −0.09, *P* = 0.69). Thus, hybrid RBR networks reduce network-level generosity because those who are positioned to provide reputational rewards to bots are less likely to give them—and, in turn, the others in the network reciprocated that lower giving behavior (the amount alter gave continued to predict giving behavior in this model, *B* = 0.38, *P* < 0.001). Importantly, this was the case even though participants were embedded in a network where they could receive reputational benefits for giving themselves. The result was lower network-level giving in hybrid systems governed by RBR.

Finally, during two rounds midway through the study (after learning what the alter to whom they could give had done, but before deciding how much to give, during rounds 4 and 8), we measured whether participants were concerned for their reputation to the same extent in the hybrid and baseline conditions. We also tested whether participants perceived bots as less worthy recipients of help, even when it was possible to receive reputational benefits for helping them. As in the token-giving models in Table 1, we controlled for round, whether the alter the ego could give to was a bot, whether the alter the ego could receive from was a bot, and the information ego received about what their alter had given in the round.

The mediation analysis models are reproduced in full in the SI. We found that participants reported being concerned for their reputation to similar extents in both conditions (Table S2, model 1, *B* = −0.06, *P* = 0.24). This was the case even among participants who were aware that their helping behavior would be reciprocated by a bot (i.e. the *hybrid condition × the alter ego receives benefits from is a bot* interaction was not significant, model 2, *B* = 0.08, *P* = 0.53). Thus, hybrid systems governed by RBR do not see lower giving because people do not care about their reputation when bots are embedded in the system, even when they are aware that their behaviors may be reciprocated by a bot.

However, being in the hybrid condition *was* associated with a significantly reduced perception that the alter to whom ego could give actually deserved help (*B* = −0.24, *P* < 0.001, see model 3 in Table S2), even after holding constant what the alter had given (which also predicted perceptions that the alter deserved help, *B* = 0.18, *P* < 0.001). Follow-up models revealed a mediated moderation process: the relationship between the hybrid condition and perceiving one's alter as deserving help was moderated by whether the alter one could help was a bot (model 4 in Table S2, *B* = −0.24, *P* < 0.05). Further, including a term for deserving help both significantly predicted token-giving (model 5 in Table S2, *B* = 0.70, *P* < 0.001) and the original significant interaction between the hybrid condition and whether the alter to whom one could give was a bot which predicted token-giving became nonsignificant (model 5, *B* = −0.38, *P* = 0.07, see also Fig. S1). Thus, the hybrid networks in our study saw reduced generosity because participants who were positioned in the network to help bots did not believe their alter deserved help, weakening network-level norms of giving even when RBR was possible.

These results demonstrate that networks involving RBR produce lower levels of giving when they are known to contain both bots and humans. Our findings reveal that this reduction in generous behavior is driven primarily by those who are in the position of helping a bot. Holding constant the alter's own behavior, those whose alter was a known bot were less generous to the alter. As a result, network-level generosity was lower in hybrid networks than in human-only ones, even though people remained concerned about their reputation in hybrid RBR networks. Instead, our results show that the reduction in giving occurred because there were weaker norms about deserving help in hybrid networks.

For the *one-shot giving study*, we analyzed 31,760 binary decisions, made by 1,985 participants (our preregistered target sample size was 2,000 participants), to give or not give a token endowment to an alter. We used generalized linear models to account for the multiple rounds of decision-making completed by each of our participants; the binary outcome was whether ego gave their one-token endowment to the alter. First, we compared giving rates in the control conditions and those RBR conditions that entailed “reputational giving,” that is, when the participant (A) could help an alter (B) in the presence of an observer (C) who could reciprocate A's behavior. (Later, we turn to the “rewarding reputations” decisions, where participants also received additional information about whether their alter had helped someone else, before deciding whether to help that alter.)

**Fig. 2.**
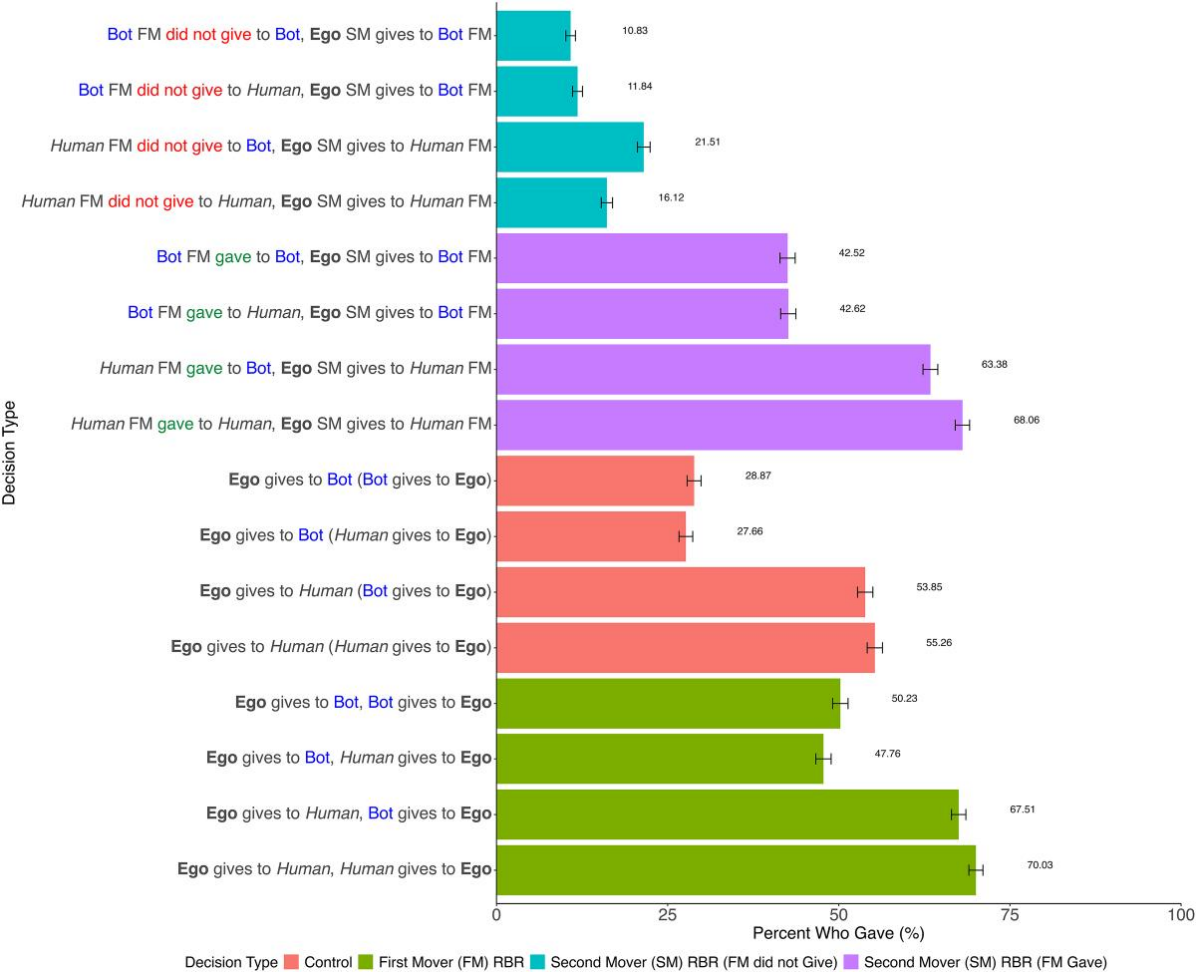
Percentage giving in the *one-shot giving study* by decision type. Error bars represent 95% CIs.

Our initial model allowed us to test (i) whether the prospect of RBR promotes giving above and beyond baseline generosity (in the control conditions) and (ii) whether, and how, the presence of bots among the role-occupants affects helping behavior. First, we find that token-giving rates were higher when ego could engage in “reputational giving”—that is, when ego's behavior toward B would be known to an observer C who could potentially help them in kind—compared with our baseline giving condition, when ego gave to B in private (odds ratio [OR] = 4.03, *P* < 0.001, Table 2, model 1). This model also reveals that, holding constant whether reputational giving was possible or not, participants were less likely to help bots than they were to help other participants (OR = 0.16, *P* < 0.001). The prospect of receiving reputational benefits reduced this difference, compared with the control conditions (Table 2, model 2, *reputational giving is possible* × *B is a bot* interaction, OR = 1.63, *P* < 0.001). This suggests that RBR enhances generosity even when some or all of the role-occupants are bots, demonstrating the robustness of reciprocity even in hybrid settings. Yet, people remained significantly more generous when they could help other participants in the network, compared with bots, as shown in Fig. 2.

**Table 2.** Token-giving when reputational giving was and was not possible, one-shot giving study.

	Model 1			Model 2			Model 3		
Predictors	OR	SE	95% CI	OR	SE	95% CI	OR	SE	95% CI
Intercept	1.20^a^	0.09	1.04–1.38	1.35^c^	0.11	1.15–1.58	1.42^c^	0.12	1.20–1.68
Reputational giving possible (RG)	4.03^c^	0.19	3.66–4.43	3.14^c^	0.25	2.68–3.67	3.35^c^	0.31	2.79–4.02
The alter that A can give to (B) is a bot	0.16^c^	0.01	0.15–0.18	0.13^c^	0.01	0.11–0.15	0.11^c^	0.01	0.10–0.14
The alter ego can receive from (C) is a bot	0.99	0.05	0.91–1.09	0.99	0.06	0.87–1.13	0.90	0.08	0.76–1.07
RG × B is a bot				1.63^c^	0.15	1.36–1.95	1.48^b^	0.19	1.15–1.91
RG × C is a bot				1.01	0.09	0.84–1.20	0.89	0.11	0.69–1.14
Bot is a bot × C is a bot							1.24	0.16	0.96–1.60
RG × Bot is a bot × C is a bot							1.21	0.22	0.85–1.74
Observations	15,880			15,880			15,880		
Marginal *R*^2^/conditional *R*^2^	0.118/0.702			0.119/0.703			0.120/0.704		
AIC	15,973.692			15,949.144			15,940.216		

^a^ *P* < 0.05.

^b^ *P* < 0.01.

^c^ *P* < 0.001.

Multilevel generalized linear models with random intercepts for multiple decision rounds nested in participants (*n* = 1,985). This model contains only the *control* and *reputational giving* conditions. Because the *rewarding reputations* conditions also entail information about what the alter had given to someone else, they are analyzed separately, in Table 3. The reference category is the control condition (where reputational giving was not possible). The outcome variable is the binary choice to give or not give a token to B.

Whether C (who could give to ego and, when reputational giving was possible, know ego's behavior and potentially reciprocate it) was a bot or a participant did not appear to impact ego's giving decision (Table 2, model 1, OR = 0.99, *P* = 0.91), which was also consistent with the chain reciprocity study. And this was the case for both the control conditions and the reputational giving condition (the interaction of *reputational giving possible × C is a bot* was not significant, model 2, OR = 1.01, *P* = 0.95, nor was there a significant *reputational giving possible × B is a bot × C is a bot* three-way interaction, model 3, OR = 1.21, *P* = 0.29).

In the *rewarding reputations* decisions, participants occupied the “C” role: they could decide whether to help an alter (A) that they knew either had or had not been generous to someone else (B). We assessed whether A's identity and B's identity had an impact on ego's behavior; in these models, we also held constant the information that ego received: i.e. whether A had helped B or not. Our main effects model revealed, unsurprisingly, that when A helped B, ego was more likely to reward A by helping them (Table 3, model 1, OR = 25.66, *P* < 0.001). Holding A's behavior constant, participants were less likely to help bots in the A position, compared with other participants (OR = 0.25, *P* < 0.001), as in the chain reciprocity study.

**Table 3.** Token-giving when rewarding reputations, one-shot giving study.

	Model 1			Model 2		
Predictors	OR	SE	95% CI	OR	SE	95% CI
Intercept	0.10^a^	0.01	0.09–0.12	0.06^a^	0.01	0.05–0.07
The alter that ego can give to (A) is a bot	0.25^a^	0.01	0.22–0.27	0.58^a^	0.07	0.46–0.73
The alter that A can give to (B) is a bot	0.99	0.05	0.90–1.09	1.73^a^	0.18	1.42–2.12
A gave their token to B	25.66^a^	1.64	22.65–29.08	61.12^a^	6.89	49.00–76.24
A is a bot × B is a bot				0.50^a^	0.08	0.36–0.68
A is a bot × A gave their token to B				0.26^a^	0.04	0.19–0.34
B is a bot × A gave their token to B				0.39^a^	0.05	0.30–0.51
A is a bot × B is a bot × A gave their token to B				2.96^a^	0.60	1.99–4.40
Observations	15,880			15,880		
Marginal *R*^2^/conditional *R*^2^	0.271/0.714			0.265/0.720		
AIC	14,153.310			14,052.677		

^a^ *P* < 0.001.

Multilevel generalized linear models with random intercepts for multiple decision rounds nested in participants (*n* = 1,985). This model contains only the *rewarding reputations* conditions. Because the *control* and *reputational giving* conditions do not have information about whether A gave their token to B, they are analyzed separately, in Table 2. The outcome variable is the binary choice to give or not give a token to A.

Whether B was a bot or not did not initially appear to impact ego's decision (OR = 0.99, *P* = 0.85). However, a model with interactions for our three key terms (whether A was a bot, whether B was a bot, and whether A gave their token to B or not) revealed that token-giving patterns varied based on their combination in a three-way interaction. We conducted simple slopes analyses along with an inspection of Fig. 2 to probe the three-way interaction. First, consider ego's decision when A did not give. If A was a *bot* that did not give, the identity of B (the alter that A did not help) did not matter: participants typically did not give to bots that did not give to bots (11% of participants gave) or to bots that did not give to humans (12%), and these were at similar levels (simple slopes analysis *P* = 0.22; see also Fig. 2). However, if A was a *human* that did not help their alter, the identity of the alter that A failed to help *did* matter: participants were more likely to give to people that did not give to bots (22%), compared with people that did not give to people (16%) (*P* < 0.01; see Fig. 2). Put differently, while people generally punished selfishness by giving at low levels, they were less likely to punish people that did not help bots.

Now, consider ego's decision when A *did* give to their alter B. Again, if A was a bot that gave, the identity of B did not matter: participants gave to bot A at similar levels when bot A helped a bot (43%), and when bot A helped a human (43%) (*P* = 0.93). But if A was a *human* that gave, the identity of B *did* matter: ego was more likely to give to a person that gave to a person (68%), compared with a person that gave to a bot (63%) (*P* < 0.01; see Fig. 2). Thus, in general, rewarding reputations did not operate as effectively when bots were embedded in the interaction: our participants did not punish other people for a lack of generosity toward bots, and did not reward people's generosity toward bots, in the same way they did toward other people.

## Discussion

As we adapt to the increasingly common addition of bots into human social networks, reciprocity in these networks may be altered in concert. Here, we asked how RBR operates to promote generosity in hybrid (i.e. human–bot) and human-only networks. Our experiments substantiate the vast array of work demonstrating that RBR promotes giving and maintains it over time (1, 3, 60, 61), even in human–bot interactions. But we also find that helping is significantly reduced when bots are embedded in the network (and, as shown in the chain reciprocity study, are *aware* that bots are embedded in the network), compared with human-only networks. The chain reciprocity study shows that this is largely driven by reduced reputational giving to bots, via people's perception that bots are less deserving of help in RBR settings.

Still further, our one-shot giving study demonstrates that *both* processes involved in RBR–reputational giving and rewarding reputations–are affected when bots occupy some or all of the roles involved. Participants in our study were significantly less likely to engage in reputational giving when they were able to help a bot (versus another person), and this was the case regardless of whether their help could be reciprocated by another participant or a bot. When they encountered bots with a good reputation, they were also less likely to reciprocate by helping them in kind, compared with humans. Perhaps, more importantly, our participants were less likely to reward *other people* who had a reputation for helping bots and were less likely to punish people who had a reputation for not helping bots. These behaviors generate the network-level reduction in generosity that we observed in our chain reciprocity study.

Taken as a whole, we build on and add to prior work showing how the introduction of bots as interaction partners can alter human prosocial behavior (26, 36, 37). Our results indicate that prosocial norms in RBR-governed interactions are weakened when known bots are present, resulting in reduced welfare among those embedded within the network. The experiments highlight the social dynamics that occur in RBR and how the introduction of bots challenges how those dynamics operate. Still further, they show that human–bot relations may affect how humans treat other humans, as when people in our experiment failed to bestow reputational rewards on those who were positioned to help a bot.

Our studies are an early step in testing RBR in hybrid versus human-only networks. As a result, they leave open several avenues for future work. For example, the bots in our study were relatively simple—they entailed the computer program and an image of a robot identified with a letter “name.” However, more complex bots, or those with more humanoid features (e.g. the ability to talk or make facial expressions, especially those displaying emotions) may be more likely to elicit prosocial behavior from humans (62, 63), including in networks governed by RBR. This is especially important because prior work has demonstrated that emotions have a distinct impact on how people assign reputations to others and foster prosocial behavior (64). Future work might test, for example, how RBR operates in hybrid networks containing more intelligent AI (like specialized large language models or embodied robots).

Relatedly, our bots were also given the simple decision rule of strong reciprocity (i.e. give what their alter had given). This choice allowed us to observe whether and how prosocial behavior in hybrid networks affects giving. However, alternative programming strategies (e.g. always contributing, never contributing) may have altered giving behavior in either positive or negative ways. Our choice to program the bots to be strong reciprocators is a relatively strong test of how the inclusion of bots in hybrid systems of humans and machines affects human behavior. But more natural designs might yoke bots' behaviors to participants' behaviors (this would also allow for experimental control without the use of deception, by matching bot behaviors to real participants' behaviors rather than ostensible participants' behaviors to bot behaviors, which we did in the chain reciprocity study), or test a variety of bot strategies to further probe how bots might affect participants' behavior in RBR settings.

Additionally, note that the networks in our experiments were fixed and, in the *chain reciprocity study* (as in related prior work (13, 65–68)), were relatively small. Still further, the human participants in both studies were made to interact with other bots or other participants as their alters, and ties could not be changed. In real-world networks, which can often be much larger than those we observed, people also can typically alter their ties, including whom they choose to help (or those they choose to ask for help). In large dynamic networks governed by RBR, humans might segregate themselves from bots as they seek to gain a positive reputation by selectively helping only humans (and rewarding other humans for helping only other humans), leaving bots to work with each other. While these dynamics might initially appear to promote giving in segregated clusters of humans and bots, it may also ultimately harm network-level productivity, if bots and humans must interact with each other to achieve their goals. These and related questions could be tested with a straightforward extension of the experiments we described here.

Moreover, the experimental control we exerted over our conditions allowed us to observe giving behavior in several types of hybrid settings. However, we did not provide participants with any information about what would happen to tokens given to bots. Prior work has demonstrated that this strategy, as well as strategies where a third-party participant receives the money that bots earn, are each associated with reduced generosity toward bots (compared with generosity toward humans) (69). Thus, while any differences in giving with bots versus humans in our studies may partly be due to the perception that bots do not need or want valuable resources, or that their earnings will go to “waste,” they cannot fully explain the differences (36). Nevertheless, providing participants with more information about the purposes of bots and what they do (including with the resources they earn) could be an important extension of the work described here, given the increasing complexity of AI with whom we must interact.

Considering how the inclusion of bots in networks shifts norms and expectations of prosocial behavior is paramount. As humans and bots of all kinds increasingly work together in these hybrid systems, how people respond to bots—both as independent agents and those working on behalf of humans—will expand and evolve. Our work shows that the social norms that guide human interactions—the propensity for humans to reward those who are generous to others, and respond to the prospect of reputational rewards with giving—can break down in the presence of bots. The benefits of hybrid systems are well known, but managing the impacts of technology on human social networks—and the concomitant norms that develop within them—should not be overlooked.

## Materials and methods

Study sessions were conducted in April through July of 2024. Participants were recruited via Prolific.com for a 15-min study, in exchange for a flat payment of $3 plus the chance to receive a bonus (ranging from $1 to $4) based on their earnings during the study. Prolific.com is a high-quality subject pool for conducting online experiments (70, 71). It is a crowdsourced convenience sample, though Prolific users are more diverse, and more representative of the United States than are typical university undergraduate samples (70). For both of our studies, participants were required to be located in the United States, be at least 18 years of age or older, and have an approval rating on Prolific of at least 98% (72). We collected their age and gender and did not analyze any other demographic information, according to our preregistration. Each study had unique participants (i.e. users were prevented from taking part in both studies).

Both studies took place online. The real-time interaction sessions for the chain reciprocity study were programmed using SMARTRIQS (73). The institutional review boards at both authors' institutions (Duke University Campus IRB, protocol nos. 2024-0419 and 2024-0487; University of Notre Dame IRB, protocol IDs 24-04-8498 and 24-05-8589), approved the research. We preregistered both studies and desired sample sizes (*chain reciprocity study*: https://aspredicted.org/5QW_BD5; *one-shot giving study*: https://aspredicted.org/WN2_JRV) before data collection began.

In both studies, we first obtained informed consent from all participants. Next, a set of instructions told participants that they would make a series of decisions with others. Their and others' decisions in one randomly chosen decision-making round (74, 75) would determine their bonus for the study. Specifically, participants were told they would receive a token endowment (worth money) in each of multiple rounds and would make a decision about giving their endowment to another player. The others in the study would be making the same decision with their own endowments. Tokens sent to someone else would be doubled, while those kept for the self were not. Thus, there was a tension between individual and collective interests: giving one's endowment would increase their value for the recipient—as a result, if all participants gave their endowments, all of them would earn more—but at the same time, keeping one's own tokens was best from any one individual's perspective.

What we call the *chain reciprocity study* assessed RBR in a series of repeated decisions in a structured network. Participants were told that they would be assigned to a group with three others with whom they would make decisions in multiple rounds. They and the others would be given an identifying letter and would occupy a position in the network, as shown in an image on their screen (see Fig. 1 for example). Arrows in the figure showed who could give tokens to whom, and the instructions explained that they and the others would make decisions in a sequential pattern: first A would make a decision about how many tokens, from 0 to 10, to send to B; then, D would learn how many tokens A sent to B before deciding how many to send to A; then, C would learn about D's decision before deciding how many tokens to send to D, and so on. Participants answered a series of quiz questions before beginning the task; missed questions were followed by a detailed description of the correct answer.

The chain reciprocity study had one key manipulation, as shown in the two columns of Fig. 1. Participants randomly assigned to the *hybrid* condition were told, before beginning the task, that their group of four would contain one other participant and two bots. Those randomly assigned to the *baseline* condition were told they would be paired with three other participants. In actuality, in both conditions participants were paired with two bots plus one other participant (participants were fully debriefed about the mild deception in the baseline condition when the study was complete, see (47–52) for more on the uses of deception in experimental research). This allowed us to hold constant the number of agents in the network whose behavior was preprogrammed and the number of agents in the network who were “real” participants making their own decisions—ensuring that our results were not driven by differences in exposure to the programmed behavior of bots.

During the task, participants viewed an image of their network with different icons to represent (known) bots versus human participants (those in the baseline condition always saw an image with four human participants). We randomly assigned the occupants of each position, as shown in Fig. 1. As a result, some of our participants were assigned to send tokens to a bot, receive tokens from a bot, or both, while our key manipulation was whether they knew bots were present in the network (the *hybrid* condition) or not (*baseline*). To keep the instructions similar across conditions, participants in the hybrid condition were not explicitly told what, if anything, would happen to earnings made by bots.

After every fourth round of the decision-making task, participants were asked a series of questions about (i) their concern for their reputation (57–59) and (ii) whether the alter to whom they could give deserved help. We intended to collect data from 12 rounds, but due to a programming error, we obtained valid data for 11 rounds (and valid reputational concern and deserving help items from two rounds, rounds 4 and 8). We asked these items at midpoints in the study rather than the end to accurately capture the causal chain from cause (condition) to mediator (reputational concern or perceptions of deserving benefits) to effect (reduced generosity) (76, 77). The SI contains additional analyses on the effects of asking mediation questions in a given round on generosity in those rounds; generally, our key results were not impacted.

Participants were not told ahead of time how many rounds there would be. They were also not told any additional information about what others in their group had done, including how many tokens the alter who could give to them had sent them, to avoid introducing income effects. In those networks where a participant was randomly assigned to the A position, in the first round they did not have information about B's decision before making their decision to give to B. However, for all other positions (and for A in each round except the first), each participant learned what the alter to whom they could give tokens had given to someone else before making their own decision.

We programmed bots to be “strong reciprocators” (53–55): after learning what their alter had sent, they sent the alter the same number of tokens. For example, if a bot's alter gave five tokens to someone else, the bot gave five tokens to the alter (if a bot was in the A role, for the first round, where no information about the alter's behavior was available, they always sent all 10 of their tokens). We chose strong reciprocation as the bots' strategy because prior work has shown that while agents programmed to engage in discriminant prosocial behavior promote generosity, agents programmed to be indiscriminately generous or indiscriminately selfish *undermine* it (56). These strategies would therefore have been a relatively weaker test of whether the presence of bots in networks governed by RBR hinders generosity. On the contrary, having our bots engage in strong reciprocity-type behavior is a more conservative test of whether, even when they engage in behavior that is known to cause prosociality, bots reduce generosity in RBR networks.

Following power analyses described in our preregistration, we aimed to collect ∼2,000 complete responses for the chain reciprocity study. After removing responses based on the criteria in our preregistration (duplicate IP addresses, reCAPTCHA scores that indicated the respondent was not human (78), and missing four or more of the seven questions on the comprehension quiz), we retained 2,192 participants (23,995 decisions) for analyses (54% female, average age = 37.6 years).

Finally, note that (regardless of condition) the chain reciprocity study entailed real-time interaction with one other participant (along with two bots) in a network. Occasionally, one of the participants in the network went “idle” (failed to make a decision about sending their tokens and clicking the button to continue after more than 90 s, *n* = 79 participants, or 3.7% of our sample, in 117 rounds). Participants who went idle were able to “come back” for subsequent rounds, and the number of rounds participants who went idle missed ranged from 1 (59 participants) to 5 (one participant). When an idle participant “came back,” they were able to click through to see the decision(s) that the computer had made on their behalf for the round(s) they missed, and could make a decision in the current round, unless it was the final round. See the Idleness in the chain reciprocity section of the SI for more analyses showing that idleness was not predicted by condition and that the results held even when excluding participants who ever went idle or controlling for idleness at the participant or group level.

On other occasions (*n* = 103 participants, or 4.7% of our sample), a participant could not be matched with another real participant after waiting for more than 3 min. When this happened, the idle or missing participant was also replaced with a bot. Bots that filled in for a missing or idle participant behaved like the other bots in the study did (i.e. engaging in “strong reciprocity,” giving 10 tokens if they were A in the first round, and perfectly reciprocating their alter's behavior for all other rounds and roles). Decisions made on behalf of idle or absent participants were not included in analyses, and participants were never aware that the other had been replaced with a bot when this happened.

In the *one-shot giving study*, participants also made decisions about sending tokens to others. However, in this study, they were told that in each round, they would be paired with new other(s) in a new interaction. They were informed that the other(s) were a mix of real other participants and bots and that the other(s) with whom they were paired in a given round would be identified to the participant as such (e.g. Participant X or Bot Y), before they made decisions. In each round, participants in the one-shot giving study received one token and made the binary decision to send it to an other, or keep it. Likewise, in each round, participants were told that another player would be making a decision about sending a token to them or not. As in the chain reciprocity study, participants completed a series of quiz questions about the task before it began; missed questions prompted a detailed explanation of the correct answer. Also in the chain reciprocity study, participants were not explicitly told what, if anything, would happen to earnings made by bots.

There were 16 different decision rounds, each of which represented a different study condition (thus, our key manipulations were within-subjects in the one-shot giving study); examples are shown in Fig. 1. First, all participants began by completing four control decisions (in random order), where the decision to help an other or not was anonymous. We measured these decisions first to capture baseline giving behavior toward participants and toward bots, before participants could be influenced by information about others' behaviors or concerns about reputation. For the control decisions, participants were told that they would give their token to either a *participant* or *bot* and were told that a different player, a *participant* or a *bot*, would be deciding whether to keep their token or give it to them at the same time. These were fully crossed, for four control decisions.

Then, participants completed 12 more rounds (in random order) that involved RBR. Four of them entailed reputational giving: participants were told that, first, they would make a decision about sending their token to an other. Then, someone else would learn whether they (the participant) had sent their token to the other before deciding whether to send their own token to the participant. As in the control decision rounds, we manipulated whether the alter to whom the participant could send their token was a *participant* or *bot* and whether the alter who could reciprocate the participant's generosity (or selfishness) was a *participant* or *bot* (fully crossed). The other eight decision rounds involved the participant rewarding reputations: they would be told whether their alter had decided to give their token to someone else and then decide whether to give to the alter. In these decisions, we crossed whether ego's alter was a *participant* or *bot*, whether the alter decided to *give* their token or *keep* it, and whether the agent the alter had helped (or not helped) was a *participant* or *bot*, for eight decision rounds total. After completing all 12 of these decisions in random order, participants answered a few open-ended questions about what made them give tokens (we used these to make decisions about dropping suspicious/poor quality responses) and the study was complete. Decisions were matched at the end of the study and participants were paid based on their real earnings in one randomly selected round.

Following power analyses described in our preregistration, we aimed to collect ∼2,000 complete responses for the one-shot giving study. After omitting responses for reasons, we preregistered (reCAPTCHA scores that indicated the respondent was not human (78), answering two or more of four quiz questions incorrectly, and suspicious or low-quality responses to at least two of three open-ended questions at the end of the study), our sample size for analyses was 1,985 participants (49% female, average age = 41.7 years).

## Supplementary Material

pgaf150_Supplementary_Data

## Data Availability

The data and scripts to replicate our results are available on the Open Science Framework: https://osf.io/973r2/. For both studies, all study conditions and data exclusions are reported in the manuscript; the full text of all measures, in the order they were presented, is given in the SI.
