# Supplementary material for: Reputation-based reciprocity in human–bot and human–human networks
Source: PNAS Nexus. 2025 May 9;4(5):pgaf150. doi: 10.1093/pnasnexus/pgaf150 (PMC12084833; doi:10.1093/pnasnexus/pgaf150)
Supplement: pgaf150_Supplementary_Data [file pgaf150_supplementary_data.docx]

**
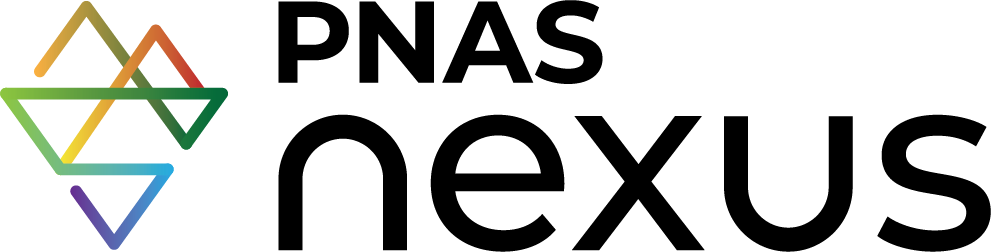
**

**Supplementary Information for**

Reputation-based reciprocity in human-bot and human-human networks

Ashley Harrell and Margaret Traeger

Corresponding author: Ashley Harrell

Email: [ashley.l.harrell@duke.edu](mailto:ashley.l.harrell@duke.edu)

**This PDF file includes:**

Supplementary text

Figures S1 to S3

Tables S1 to S23

SI References

Supplementary Text

**Selected text from the study instructions and all study survey items.**

*All study conditions are reported in the manuscript; the full text of all measures, in the order that they were presented to participants, is shown and described below.*

*Chain reciprocity study*

*[Basic instructions]*

Welcome! In this study you will complete a decision-making task in a group of four. Your group of four will consist of [*you, one real other participant, and two bots*; *you and three other participants*].

The decision-making task has multiple rounds. At the start of each round, you and each of the others will receive 10 tokens (each token is worth 10 cents). You will take turns indicating how many tokens you want to keep for yourself, and how many you want to send to another group member. Decisions will be made in sequential order.

Any tokens you send to an other will be doubled. Similarly, any tokens sent to you will be doubled. Your total earnings per round are the doubled number of tokens you received (if you received any), plus whatever tokens you kept for yourself (if you kept any).

Your bonus payment for today’s study, which you can earn if you do not go idle during the real-time task, will be $1 PLUS your earnings from one randomly selected round of the task. This means that all rounds are important, as any given round may determine what your final bonus will be. If you do not go idle during the task, your bonus can range from $1 to $4.

Each group member will be identified by a letter (e.g., Participant A or [*Bot*, *Participant*] B), which will stay the same throughout the task. Group members may send tokens to each other as shown in the example image below. The arrows show who can send tokens to whom, and this will also stay the same throughout the task.


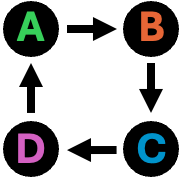


You and the others will make decisions in a counter-clockwise pattern:

A goes first, then D, then C, then B.

Specifically, on Round 1,

- A begins by deciding how many tokens to send to B.
- Next, D will learn how many tokens A sent to B before deciding how many tokens to send to A.
- Then, C will learn how many tokens D sent to A before deciding how many tokens to send to D.
- Finally, B will learn how many tokens C sent to D before deciding how many tokens to send to C.

After this, all group members will have made a decision for the round; the next round will begin. At the beginning of Round 2, A will learn how many tokens B sent to C before deciding how many tokens to send to B. And so on - the process will continue for several rounds. Aside from what the group member ahead of them did, group members will not receive information about what others did between rounds (including how many tokens were sent to them).

Make sure you have read the instructions carefully. Next, you will answer some quiz questions to check your understanding.

*[Quiz questions. Each question was presented on an individual screen, along with the same image of the chain shown above. Answers were followed by a Correct/Incorrect message and a brief explanation of the answer.]*

Imagine you are A. To whom can you send tokens? You may refer to the image above, if you need to.

- B
- B and D
- B, C, and D

Again, imagine you are A. Before you send tokens to B, you will be told:

- How many tokens D sent to you
- How many tokens B sent to C
- How many tokens everyone else sent

Again, imagine you are A. Before D sends tokens to you, D will be told:

- How many tokens C sent to them
- How many tokens you sent to B
- How many tokens B sent to C
- How many tokens everyone else sent

Again, imagine that you are A. You send all 10 of your 10 tokens to B. Then, D sends all 10 of their 10 tokens to you. How many tokens do you end with this round? (Remember, tokens sent to an other are doubled, while tokens kept for the self are not.)

- 0
- 10
- 20
- 30

Again, imagine that you are A. You send 0 of your 10 tokens to B. Then, D sends all 10 of their 10 tokens to you. How many tokens do you end with this round? (Remember, tokens sent to an other are doubled, while tokens kept for the self are not.)

- 0
- 10
- 20
- 30

Again, imagine that you are A. You send 0 of your 10 tokens to B. Then, D sends 0 of their 10 tokens to you. How many tokens do you end with this round? (Remember, tokens sent to an other are doubled, while tokens kept for the self are not.)

- 0
- 10
- 20
- 30

Again, imagine that you are A. You send 10 of your 10 tokens to B. Then, D sends 0 of their 10 tokens to you. How many tokens do you end with this round? (Remember, tokens sent to an other are doubled, while tokens kept for the self are not.)

- 0
- 10
- 20
- 30

*[Task content. Sample image shown based on a participant randomly assigned to the* hybrid *condition, in the B position, with bots randomly assigned to the C and D positions. Other participants saw different images, depending on their randomly assigned condition, structure, and position in the chain.]*

Your group ([*you, one real other participant, and two bots*; *you and three other participants*]) is shown below; you are **Participant B.**


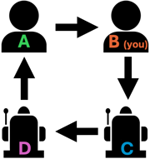


**Bot C** went before you and sent **1** tokens to **Bot D.** (Since tokens sent are doubled, they became **2** tokens.)

**How many tokens do you want to send** to **Bot C this round?** Enter a number between 0 and 10.

(Remember, **Participant A** will learn how many tokens you sent **Bot C** before deciding how many tokens to send you.)

*[Mediation questions asked after rounds 4, 8 and 12^^[[1]](#footnote-1)^^]*

*[Reputational concern (5 point response scale, ranging from 1 (“Totally Disagree”) to 5 (“Totally Agree”))]*

Before deciding how many tokens to send **Bot C**, please answer the following questions

I am thinking about what **Participant A** will think about me

It’s important to me that **Participant A** has a positive evaluation about me

It is important to me that **Participant A** accepts me

I am not considering what **Participant A** would say about me

*[Deserving Help (5 point response scale, ranging from 1 (“Totally Disagree”) to 5 (“Totally Agree”))]*

Before deciding how many tokens to send **Bot C**, please answer the following questions

**Bot C** deserves tokens this round

**Bot C** should receive tokens this round

**Bot C** ought to be treated the same way they treat others^^[[2]](#footnote-2)^^

*[Suspicion check question(s) asked at the end of the study. All participants answered the first question, with those in the baseline condition asked about “the [multiple] other participants” and those in the hybrid condition asked about “the other participant”. Only those in the hybrid condition answered the second question.]*

During the decision-making task, I thought the other participant [other participants] in my group was [were]… [definitely not a real person [definitely not real people] – definitely a real person [definitely real people]

During the decision-making task, I thought the bots in my group were… [definitely not real people – definitely real people]

*One-shot giving study*

*[Pre-study survey. Participants first completed a standard SVO scale before reading the study instructions. We did not pre-register analyses about this scale and intended to use it for a separate project; it is not analyzed here.]*

In this set of survey questions, we ask you to imagine that you have been randomly paired with another person, whom we will refer to simply as the “other.” Other is someone you do not know and that you will not knowingly meet in the future. Both you and Other will be making choices between different options. Your own choices will produce points for yourself and Other. Likewise, Other’s choice will produce points for them and for you. Every point has value: The more points you receive, the better for you, and the more points Other receives, the better for them.

**Here’s an example of how this task works.**

A. You get 500, Other gets 100
B. You get 500, Other gets 500
C. You get 550, Other gets 300

In this example, if you chose A you would receive 500 points and Other would receive 100 points; if you chose B, you would receive 500 points and Other 500; and if you chose C, you would receive 550 points and Other 300. So, you see that your choice influences both the number of points you receive and the number of points the other receives.

Before you begin making choices, keep in mind that there are no right or wrong answers – choose the option that you, for whatever reason, prefer most. Also, remember that the points have value: The more of them you accumulate, the better for you. Likewise, from the Other’s point of view, the more points they accumulate, the better for them.

Click the button below when you are ready to begin these survey questions.

Choose the option you prefer the most:

You get 480; Other gets 80

You get 540; Other gets 280

You get 480; Other gets 480

Choose the option you prefer the most:

You get 560; Other gets 300

You get 500; Other gets 500

You get 500; Other gets 100

Choose the option you prefer the most:

You get 520; Other gets 520

You get 520; Other gets 120

You get 580; Other gets 320

Choose the option you prefer the most:

You get 500; Other gets 100

You get 560; Other gets 300

You get 490; Other gets 490

Choose the option you prefer the most:

You get 560; Other gets 300

You get 500; Other gets 500

You get 490; Other gets 90

Choose the option you prefer the most:

You get 500; Other gets 500

You get 500; Other gets 100

You get 570; Other gets 300

Choose the option you prefer the most:

You get 510; Other gets 510

You get 560; Other gets 300

You get 520; Other gets 110

Choose the option you prefer the most:

You get 550; Other gets 300

You get 500; Other gets 100

You get 500; Other gets 500

Choose the option you prefer the most:

You get 480; Other gets 100

You get 490; Other gets 490

You get 540; Other gets 300

*[Basic instructions]*

In this study, you will make a series of decisions about distributing tokens to other players. Some of these players will be real other participants, and others will be bots.

The basic directions are as follows: you and the other players in this study will be identified during the task with a random identifying letter (e.g., Participant X or Bot Y).

Your choices, and the choices of the other players, will be connected after the study is complete, and will determine your (and others’) bonus payment for the study.

That is, your bonus payment will depend not only on the choices that you make but also on the choices that others make. Similarly, others' bonus payments will depend not only on the choices that they make but also the choices that you (and others) make.

Specifically, the study consists of several decision rounds.

At the beginning of each decision round, you will receive a token, worth one dollar. You will be asked to indicate if you would like to *give* your token to another player, or if you would like to *keep* it for yourself.

Similarly, the other players in this study (which, again, will be a mix of real other participants and bots) will receive a token in each round, and will also be deciding if they would like to give it to another player (including, possibly, you), or keep it for themselves.

Your decisions, and the other players' decisions, will determine the bonus you will earn.

Importantly, if you *give* your token to another player, it will double, to become two tokens. (However, if you *keep* your token for yourself, it will *not* be doubled.)

Similarly, if another player *gives* their token to you, it will double, and become two tokens.

In each round, you will decide if you want to keep or give your token to one other player (e.g., Participant X or Bot Y). Likewise, a *different* other player (e.g., Participant A or Bot B) will be assigned to decide if they will keep or give their token to you. You will make decisions with different others in each round.

Your final earnings in each round will consist of:

1) the token you kept for yourself, if you decided to keep, plus
2) the doubled tokens an other gave to you, if they decided to give.

Tokens are worth $1 each and you can end a round with anywhere from 0 to 3 tokens.

You start with an automatic $1 bonus. This means that the minimum bonus you could earn in a round is $1 (you end the round with 0 tokens - e.g., you give your token to a player, but another player does not give their token to you) and the maximum bonus you could earn in a round is $4 (you end the round with 3 tokens – you keep your token rather than giving it to a player, and another player gives their token to you, which doubles to become 2 tokens).

Your bonus will be your earnings from one randomly selected round. This means that *all* rounds are important, for both you and others.

Make sure you have carefully read and understand the instructions so far. Next, you will answer some questions to check your understanding.

*[Quiz questions. Each question was presented on an individual screen. Answers were followed by a Correct/Incorrect message and a brief explanation of the answer.]*

Imagine you decide to give your token to a player. Another player gives their token to you.

How many tokens do you end this round with?

- 0 tokens
- 1 token
- 2 tokens
- 3 tokens

Imagine you decide to keep your token, rather than giving it to a player. Another player gives their token to you.

How many tokens do you end this round with?

- 0 tokens
- 1 token
- 2 tokens
- 3 tokens

Imagine you decide to give your token to a player. Another player keeps their token, rather than giving it to you.

How many tokens do you end this round with?

- 0 tokens
- 1 token
- 2 tokens
- 3 tokens

Imagine you decide to keep your token, rather than giving it to a player. Another player keeps their token, rather than giving it to you.

How many tokens do you end this round with?

- 0 tokens
- 1 token
- 2 tokens
- 3 tokens

*[Additional set of instructions before task]*

At the beginning of each decision round, we will let you know the identifying letter (e.g., Participant X, Bot Y) of the player to whom you may give tokens in the round. Remember, you will never interact with the same other player more than once.

Importantly, we may also give you some additional information. Therefore, make sure you continue to carefully read the instructions and information given before each decision round, because this information will change.

[*Task content*]

*[Control decision for the* Bot, Participant *decision-round shown; one of four decision types. The set of four control decisions were always presented first, but in random order (Bot, Bot; Participant, Participant; Participant, Bot) and in each one, the others’ identifying letters were different and its corresponding image was shown to the participant.]*

**For this round, you will decide whether to *keep* your token, or *give* it to Bot P.**


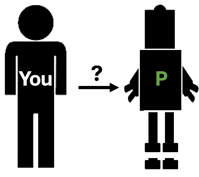


Once you make your decision, that will be the end of this round. **Bot P will not learn of your decision, nor will anyone else.**

**A different** player, **Participant F**, will be deciding whether to keep or give their token to you.


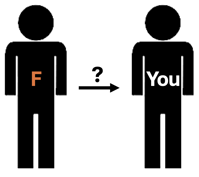


Click the button below when you are ready to begin.

**Would you like to give your token to Bot P, or keep it?**

(Remember, **Participant F** will be deciding whether or not to give their token to you.)

Give to **Bot P**

Keep

We will now begin the next round. Identifying letters have been reassigned, and you will be paired with players with whom you have not yet interacted.

*[First mover decisions. Sample decision shown for the* Bot, Bot *decision-round. There were three other first mover decisions presented in random order (Participant, Bot; Participant, Participant; Bot, Participant) and in each one, the identifying letter was different and its corresponding image was shown on the screen]*

**For this round, you will decide whether to *keep* your token, or *give* it to Bot G. Then, another player, Bot Q, will decide whether to keep their token, or give it to *you*.**

You will make decisions as follows. ***You* go first.**


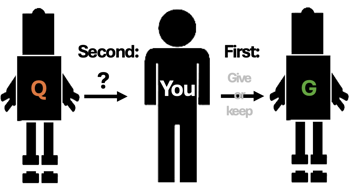


Specifically:

**First,** *you* decide if you will give your token to **Bot G**.

**Next**, **Bot Q learns whether or not you gave your token to** **Bot G**. **Then, Bot Q** **decides whether or not to give their token to *you*.**

Click the button below when you are ready to begin.

**Would you like to give your token to Bot G, or keep it?**

(Remember, **Bot Q** will learn whether or not you gave your token to **Bot G**. Then, **Bot Q** will decide whether or not to give their token to you.)

Give to **Bot G**

Keep

*[Second mover decisions. Sample decision shown for the* Bot, Participant, First mover gave *decision-round. There were seven other second mover decisions presented in random order (Participant, Bot, First mover gave/didn’t give; Participant, Participant, First mover gave/didn’t give; Bot, Bot, First mover gave/didn’t give) and in each one, the identifying letter was different and its corresponding image was shown on the screen]*

**For this round, another player, Participant B, will decide whether to *keep* their token, or *give* it to Bot W. Then, you will decide whether to keep your token, or give it to Participant B.**

You will make decisions as follows. **Participant B goes first.**


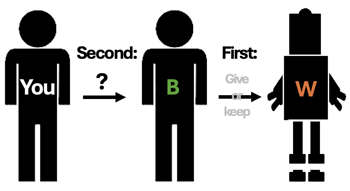


Specifically:

**First,** **Participant B** decided if they will give their token to **Bot W**.

**Next**, ***you* learn whether or not Participant B gave their token to** **Bot W**.

**Then, *you*** **decide whether or not to give your token to Participant B.**

Click the button below when you are ready to begin.

**Participant B** decided to GIVE their token to **Bot W**.

Therefore, **Bot W** received 2 tokens from **Participant B.**

**Would you like to give your token to Participant B, or keep it?**

Give to **Participant B**

Keep

*[Post study open-ended questions. We used these to check for (and omit) suspicious/non-sensical responses to at least two of the three items.]*

Please answer the questions below. You may be brief and write informally (e.g. complete sentences are not necessary.)

What do you think made Bots decide to give (vs. keep) tokens? We are interested in any thoughts you have on why Bots made the choices they did.

What do you think made Participants decide to give (vs. keep) tokens? We are interested in any thoughts you have on why Participants made the choices they did.

What made You give tokens when you gave them, or keep them when you kept them? Did it matter if the recipient was a participant or a bot? We are interested in any thoughts you have on why you made the choices you did.

Sample demographics

**Table S1. Sample demographics, both studies**

|  |  | **Chain reciprocity study** | **One-shot giving study** |
| --- | --- | --- | --- |
| **N_participants_** |  | 2,192 | 1,985 |
| **Age (mean (SD))** |  | 37.6 (12.1) | 41.7 (12.6) |
|  | *Missing (N)* | 11 | 15 |
| **Sex (%)** | *Female* | 1182 (54.0) | 979 (49.3) |
|  | *Male* | 986 (44.8) | 982 (49.5) |
|  | *Prefer not to say* | 11 (0.5) | 7 (0.4) |
|  | *Missing* | 13 (0.6) | 17 (0.9) |

**Mediation analyses discussed in the main text**

**Table S2. Mediation analysis, *chain reciprocity study***

|  | **Model 1:**  **RC** | | **Model 2:**  **RC** | | **Model 3:**  **DH** | | **Model 4:**  **DH** | | **Model 5: Token-giving** | |
| --- | --- | --- | --- | --- | --- | --- | --- | --- | --- | --- |
| *Predictors* | *Est.* | *SE* | *Est.* | *SE* | *Est.* | *SE* | *Est.* | *SE* | *Est.* | *SE* |
| Intercept | 3.14 ^***^ | 0.09 | 3.19 ^***^ | 0.12 | 2.86 ^***^ | 0.08 | 2.84 ^***^ | 0.10 | 1.10 ^***^ | 0.25 |
| Hybrid condition (H) | -0.06 | 0.05 | -0.16 | 0.16 | -0.24 ^***^ | 0.04 | -0.19 | 0.13 | 0.19 | 0.27 |
| The alter that ego can *give to* is a bot | 0.02 | 0.06 | -0.01 | 0.09 | -0.11 ^*^ | 0.05 | 0.01 | 0.07 | 0.14 | 0.15 |
| The alter that ego can *receive from* is a bot | -0.12 | 0.06 | -0.16 | 0.09 | -0.00 | 0.05 | -0.09 | 0.07 | 0.07 | 0.15 |
| Round | -0.01 ^*^ | 0.00 | -0.01 ^*^ | 0.00 | -0.01 ^*^ | 0.00 | -0.01 ^*^ | 0.00 | -0.01 | 0.01 |
| Amount Alter_give to_ gave their alter this round | 0.02 ^***^ | 0.00 | 0.02 ^***^ | 0.00 | 0.18 ^***^ | 0.00 | 0.18 ^***^ | 0.00 | 0.49 ^***^ | 0.01 |
| H x Alter_give to_ is a bot |  |  | 0.06 | 0.12 |  |  | -0.24 ^*^ | 0.10 | -0.38 | 0.21 |
| H x Alter_receive from_ is a bot |  |  | 0.08 | 0.12 |  |  | 0.16 | 0.10 | -0.12 | 0.21 |
| Alter_give to_ deserves help scale |  |  |  |  |  |  |  |  | 0.70 ^***^ | 0.04 |
| Observations | 4384 | | 4384 | | 4384 | | 4384 | | 4331 | |
| Marginal R^2^ / Conditional R^2^ | 0.008 / 0.871 | | 0.008 / 0.871 | | 0.288 / 0.764 | | 0.292 / 0.764 | | 0.489 / 0.729 | |
| AIC | 11000.446 | | 11008.872 | | 11265.413 | | 11261.312 | | 19262.604 | |
| ** p<0.05 ** p<0.01 *** p<0.001* | | | | | | | | | | |

**Note:** Multilevel linear models with random intercepts for the two decision-rounds (rounds 4 and 8) nested in (N = 2,192) participants. Model 3 and 4 also include random intercepts for the network (N = 1,293 networks with one real other participant); including random intercepts at the network level for the other models resulted in a singular fit. The outcome variable for Models 1-2 and 3-4 is average *reputational concern* (RC) and *deserving help* (DH) scales respectively, which could range from 1 to 5; for Model 5, the outcome variable is the number of tokens ego gave their alter, ranging from 0 to 10.

*
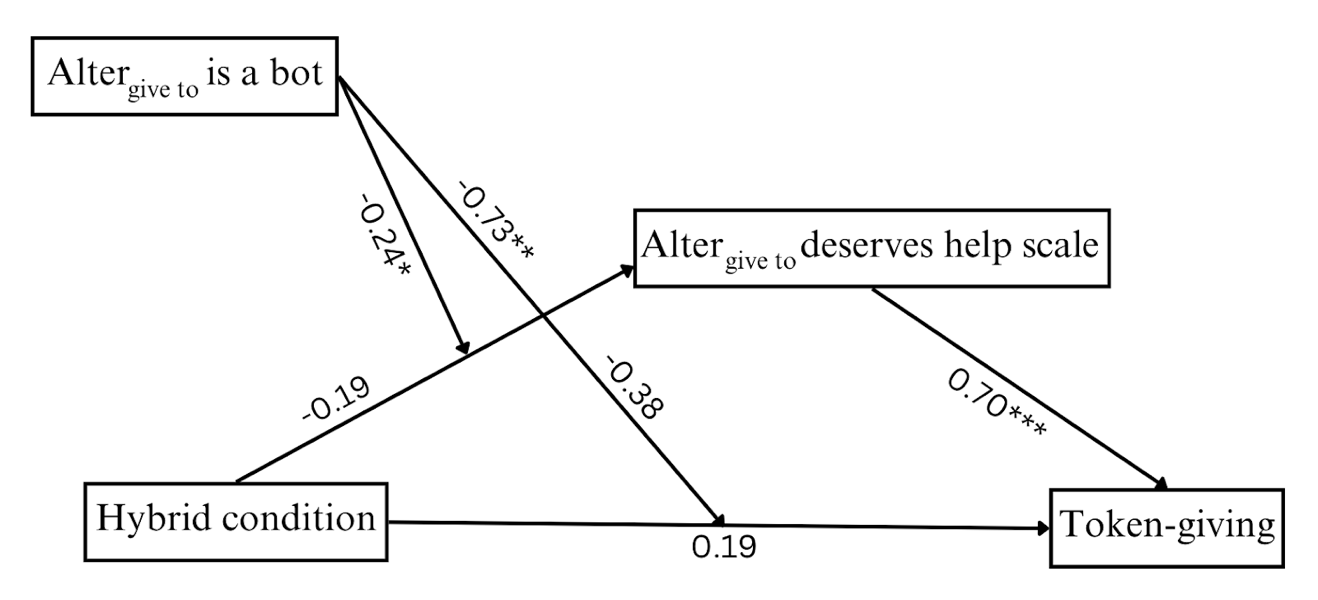
*

Fig. S1. Mediated moderation analysis, *chain reciprocity study*

**Note:** Diagram showing the mediated moderation process. The hybrid condition *x* alter_give to_ is a bot interaction significantly predicts token-giving when the deserving help scale is not included in the model (Table 1 in the main text, Model 2, *B* = -0.73, *p* < 0.01) but becomes nonsignificant when deserving help is included in the model (Table S2, Model 5, *B* = -0.38, *p* = 0.07). **p<0.05 **p<.01 ***p<.001*

**Exploring behavior over time**

*Decision-making by first movers*

In the chain reciprocity study*,* participants made repeated decisions with the same alters in a series of rounds. We conducted supplementary analyses to see if giving patterns differed across the rounds of the study, for several reasons. First, as discussed in the main text, we had to program our bots’ decisions. This created path dependencies over the repeated rounds of decision-making, where the bots’ (programmed) decision rule, and not our experimental manipulations alone, were impacting participant behaviors that may explain our results. We therefore conducted supplementary analyses of our participants’ decisions in the very *first* round of interaction, before bots’ behaviors could have a chance to impact participants’ behaviors at all.

This analysis included only the decisions of participants who were assigned to the A position (i.e., those who were the first decision-maker in the “chain”, when a human participant was randomly assigned to that position) and in only the first round of decision-making (before they could learn anything about what B had given in the previous round, which A learned in all rounds except the first; those assigned to all other positions learned information about what the alter in front of them had done in all rounds *including* the first). Looking at these first movers’ decisions in this first round allows us to assess if just the *anticipation* of interacting with bots affects giving in RBR networks, before the bots’ behaviors can play any role in their decision-making. On another note, these first mover decisions were dropped from all our other analyses (because our other models control for the information ego received about what their alter had given, and first movers did not receive any information), so analyzing their decisions here also allows us to examine our dropped data.

Model 1 in Table S3 is equivalent to Model 1 shown in Table 1 of the main text, but only for first movers in the first round. The only difference is that the models here do not control for round or what the alter gave in this round, because analyses were conducted only on the first round and there was no information about what alter gave. Results reveal that first movers in the hybrid condition gave less than first movers in the baseline condition, even though they had not yet been exposed to any bot (or participant) behaviors (B = -0.91, *p* < 0.001).

Model 2 contains the interaction between being in the hybrid condition and the identity of the alter to whom the first mover could give. Model 3 also contains the interaction between being in the hybrid condition and the identity of the alter who could (potentially) reward ego for giving. Model 2 is the best fit according to the AIC and chi-square goodness-of-fit tests. It shows the same interaction we identified in the main text: the difference in generosity between the hybrid and baseline condition was driven by those who were positioned to give to a bot—they gave significantly less to bots than they did to participants (*B* = -1.23, *p* = 0.04). While this interaction becomes nonsignificant in Model 3 (where we added an additional, non-significant interaction), we suspect it is a power issue due to the very small sample size in the first-mover models. As in our main models, the identity of the alter who could reward ego’s reputation did not impact giving decisions.

**Table S3. Token-giving in the *chain reciprocity study*, first movers only**

|  | **Model 1** | | | **Model 2** | | | **Model 3** | | |
| --- | --- | --- | --- | --- | --- | --- | --- | --- | --- |
| *Predictors* | *Est.* | *SE* | *95% CI* | *Est.* | *SE* | *95% CI* | *Est.* | *SE* | *95% CI* |
| Intercept | 7.96 ^***^ | 0.43 | 7.11 – 8.81 | 7.56 ^***^ | 0.47 | 6.63 – 8.49 | 7.63 ^***^ | 0.58 | 6.48 – 8.77 |
| Hybrid condition (H) | -0.91 ^***^ | 0.26 | -1.41 – -0.40 | 0.03 | 0.53 | -1.01 – 1.07 | -0.08 | 0.81 | -1.68 – 1.52 |
| The alter that ego can *give to* is a bot | -0.37 | 0.32 | -1.00 – 0.26 | 0.20 | 0.43 | -0.63 – 1.04 | 0.18 | 0.44 | -0.67 – 1.04 |
| The alter that ego can *receive from* is a bot | -0.30 | 0.31 | -0.91 – 0.30 | -0.34 | 0.31 | -0.94 – 0.26 | -0.40 | 0.46 | -1.30 – 0.50 |
| H x Alter_give to_ is a bot |  |  |  | -1.23 ^*^ | 0.60 | -2.42 – -0.04 | -1.19 | 0.64 | -2.45 – 0.07 |
| H x Alter_receive from_ is a bot |  |  |  |  |  |  | 0.11 | 0.62 | -1.10 – 1.33 |
| Observations | 579 | | | 579 | | | 579 | | |
| R^2^ / R^2^ adjusted | 0.023 / 0.018 | | | 0.030 / 0.024 | | | 0.031 / 0.022 | | |
| AIC | 2948.792 | | | 2946.636 | | | 2948.601 | | |
| ** p<0.05 ** p<0.01 *** p<0.001* | | | | | | | | | |

**Note:** Linear regressions predicting the number of tokens, ranging from 0 to 10, that first movers (i.e., participants randomly assigned to the A position, on the first round of interaction) gave to their alter.

*Decision-making patterns over time, by condition*

We also examined whether the patterns we described in the main text unfolded differently over time, across the rounds of the study. While the models in the main text *control* for round, we conducted follow-up analyses *interacting* round with our key manipulation, the hybrid (versus baseline) condition, in both the main model (Model 1 in Table S4, which corresponds to Model 1 in Table 1 of the main text), as well as the model with interactions between the hybrid condition and being positioned such that one could give to, and/or receive from, a bot (Model 2 in Table S4, which corresponds to Model 2 in Table 1 of the main text). Model 3 below includes the three-way interactions between the conditions and round. As shown in Table S4, including the interactions with round did not alter the findings described in the main text; additionally, none of them were significant. This suggests that the patterns we observed were relatively steady across the rounds of the study session.

**Table S4. Key models, *chain reciprocity study*, with interactions by round**

|  | **Model 1** | | | **Model 2** | | | **Model 3** | | |
| --- | --- | --- | --- | --- | --- | --- | --- | --- | --- |
| *Predictors* | *Est.* | *SE* | *95% CI* | *Est.* | *SE* | *95% CI* | *Est.* | *SE* | *95% CI* |
| Intercept | 5.11 ^***^ | 0.18 | 4.76 – 5.45 | 4.79 ^***^ | 0.23 | 4.33 – 5.25 | 4.81 ^***^ | 0.25 | 4.32 – 5.30 |
| Hybrid condition (H) | -0.63 ^***^ | 0.11 | -0.84 – -0.41 | -0.01 | 0.32 | -0.64 – 0.62 | -0.04 | 0.34 | -0.71 – 0.63 |
| Round (R) | 0.01 | 0.01 | -0.00 – 0.02 | 0.01 | 0.01 | -0.00 – 0.02 | 0.01 | 0.02 | -0.04 – 0.05 |
| The alter that ego can *give to* is a bot | -0.17 | 0.12 | -0.39 – 0.06 | 0.21 | 0.17 | -0.11 – 0.54 | 0.24 | 0.18 | -0.11 – 0.60 |
| The alter that ego can *receive from* is a bot | -0.08 | 0.12 | -0.30 – 0.15 | -0.03 | 0.17 | -0.35 – 0.30 | -0.08 | 0.18 | -0.44 – 0.28 |
| Amount Alter_give to_ gave their alter this round | 0.36 ^***^ | 0.01 | 0.34 – 0.37 | 0.36 ^***^ | 0.01 | 0.34 – 0.37 | 0.36 ^***^ | 0.01 | 0.34 – 0.37 |
| H x R | 0.01 | 0.01 | -0.01 – 0.03 | 0.01 | 0.01 | -0.01 – 0.03 | 0.02 | 0.03 | -0.04 – 0.08 |
| H x Alter_give to_ is a bot |  |  |  | -0.73 ^**^ | 0.23 | -1.19 – -0.28 | -0.76 ^**^ | 0.25 | -1.25 – -0.26 |
| H x Alter_receive from_ is a bot |  |  |  | -0.10 | 0.23 | -0.55 – 0.36 | -0.03 | 0.26 | -0.53 – 0.47 |
| R x Alter_give to_ is a bot |  |  |  |  |  |  | -0.01 | 0.02 | -0.04 – 0.03 |
| R x Alter_receive from_ is a bot |  |  |  |  |  |  | 0.01 | 0.02 | -0.02 – 0.05 |
| H x R x Alter_give to_ is a bot |  |  |  |  |  |  | 0.00 | 0.02 | -0.04 – 0.05 |
| H x R x Alter_receive from_ is a bot |  |  |  |  |  |  | -0.01 | 0.02 | -0.06 – 0.03 |
| Observations | 23416 | | | 23416 | | | 23416 | | |
| Marginal R^2^ / Conditional R^2^ | 0.175 / 0.661 | | | 0.178 / 0.661 | | | 0.178 / 0.661 | | |
| AIC | 100721.887 | | | 100716.000 | | | 100748.681 | | |
| ** p<0.05 ** p<0.01 *** p<0.001* | | | | | | | | | |

**Note:** Multilevel linear models with random intercepts for the multiple decision-rounds nested in participants (N = 2,192) nested in networks (N = 1,293) with one real other participant and, because the models include cross-level interactions [(1)](https://www.zotero.org/google-docs/?3pAOWE), random slopes for decision-round. Round ranges from 0 to 10. The first round decisions for participants in the A position are dropped because they did not receive information about the amount their alter had given in the first round, by necessity. The outcome variable is the number of tokens ego gave their alter, ranging from 0 to 10.

*The impact of assessing our mediators at mid-points during the study*

Finally, as reported in the main text, at several mid-points during the study (after ego learned what their alter had given someone else, but before deciding how much to give to that alter, during rounds 4 and 8) we measured whether participants were concerned for their reputation to the same extent in the hybrid and baseline conditions. We also tested whether participants perceived their alter as a less worthy recipient of help when they were aware the alter was a bot, even when it was possible to receive reputational benefits for helping them. While asking participants these items prior to making a token-giving decision, rather than at the end of the study, allows us to accurately capture the causal chain from cause (condition) to mediator (reputational concern or perceptions of deserving benefits) to effect (reduced generosity) [(2, 3)](https://www.zotero.org/google-docs/?M4Ghfj), it also runs the risk of affecting participants’ subsequent behaviors in the repeated decision-making task. For example, participants may have been more generous in the rounds where they answered the mediator questions, because reputational concern or perceptions that the alter deserves help may have been more salient to them immediately after answering them.

A visual inspection of token-giving patterns across the rounds, shown in Figure S2, suggests that there may have been a temporary increase in token-giving in the rounds where we assessed our mediators (i.e., during rounds 4 and 8). As a result, we also conducted supplementary models that contained the same terms as those in the main text, but with an additional variable controlling for whether or not the mediator items were asked in the round.

**
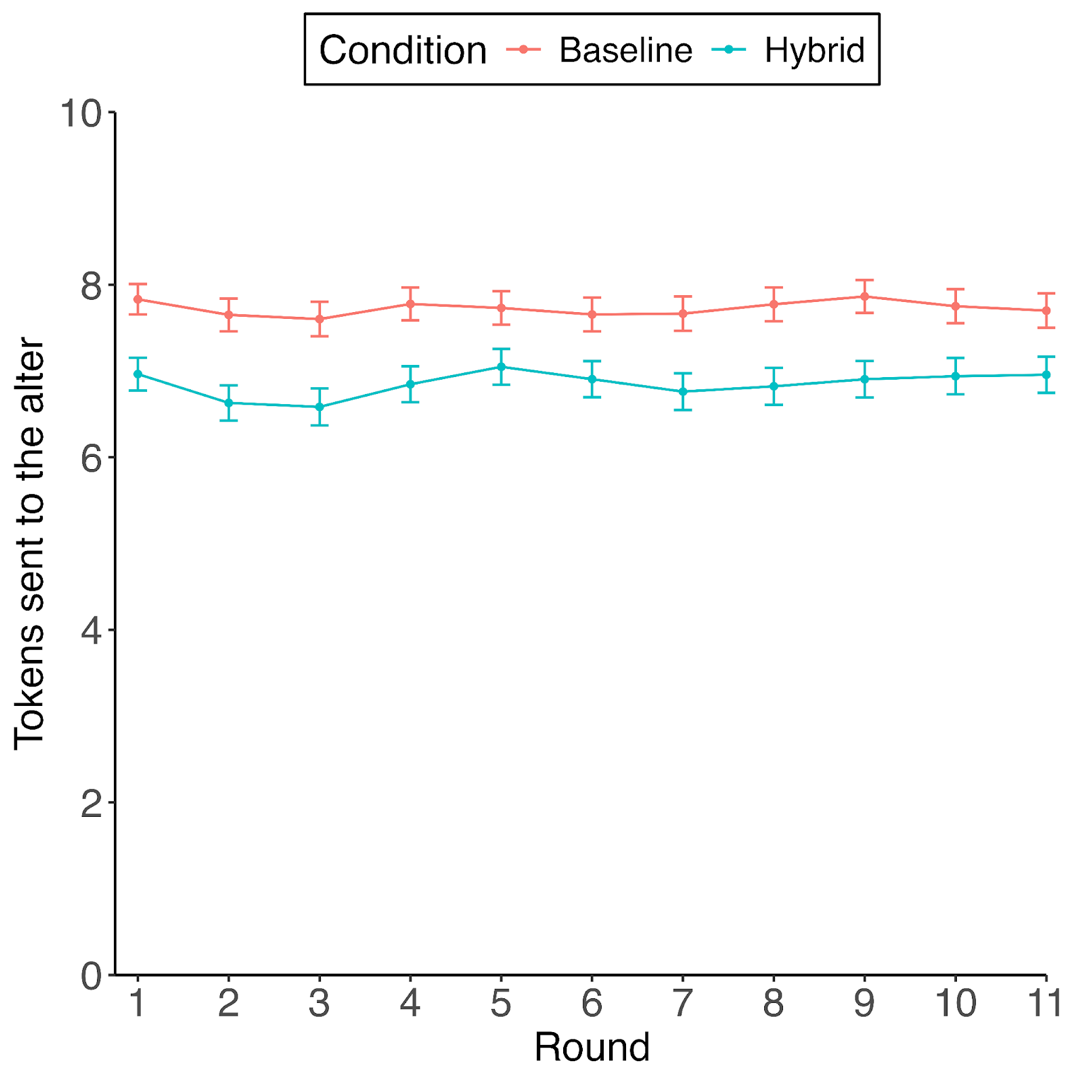
**

**Figure S2. Token-giving patterns, *chain reciprocity study*, over time**

**Note:** Error bars represent 95% confidence intervals.

Model 1 in Table S5 is equivalent to Model 1 in Table 1 in the main text, but with the additional term for whether the mediator questions were asked in the round. It shows that token-giving was higher in those rounds where the mediator items were asked (B = 0.08, *p* < 0.05). However, the effect of the hybrid condition (our key finding) remained significant (B = -0.54, *p* < 0.001). Model 2 shows that the hybrid condition *x* the alter ego can give to is a bot interaction remained significant as well (B = -0.73, *p* < 0.01). Finally, Model 3 examines the three-way interaction between the hybrid condition, whether the alter ego can give to is a bot, and the new term for whether mediators were asked in the round. None of the interactions with the *asked mediators this round* item were significant, and the *hybrid condition x the alter ego can give to is a bot* interaction (i.e., our key result) held (B = -0.75, *p* < 0.01). This means that, while asking the mediator items did appear to cause a short-term increase in giving behavior immediately after answering them, this effect occurred similarly across our conditions, regardless of whether participants were in the hybrid or baseline condition, or positioned to give to or receive from a bot (or not).

**Table S5. Key models, *chain reciprocity study*, assessing the effects of asking the mediator items**

|  | **Model 1** | | | **Model 2** | | | **Model 3** | | |
| --- | --- | --- | --- | --- | --- | --- | --- | --- | --- |
| *Predictors* | *Est.* | *SE* | *95% CI* | *Est.* | *SE* | *95% CI* | *Est.* | *SE* | *95% CI* |
| Intercept | 4.83 ^***^ | 0.17 | 4.50 – 5.16 | 4.52 ^***^ | 0.23 | 4.08 – 4.97 | 4.53 ^***^ | 0.23 | 4.08 – 4.97 |
| Hybrid condition (H) | -0.54 ^***^ | 0.10 | -0.73 –  -0.35 | 0.07 | 0.31 | -0.54 – 0.67 | 0.07 | 0.31 | -0.54 – 0.67 |
| Round | 0.02 ^***^ | 0.00 | 0.01 – 0.02 | 0.02 ^***^ | 0.00 | 0.01 – 0.02 | 0.02 ^***^ | 0.00 | 0.01 – 0.02 |
| The alter that ego can *give to* is a bot | -0.17 | 0.11 | -0.39 – 0.05 | 0.21 | 0.16 | -0.11 – 0.53 | 0.21 | 0.16 | -0.11 – 0.53 |
| The alter that ego can *receive from* is a bot | -0.07 | 0.11 | -0.29 – 0.15 | -0.03 | 0.16 | -0.35 – 0.30 | -0.04 | 0.17 | -0.36 – 0.28 |
| Amount Alter_give to_ gave their alter this round | 0.38 ^***^ | 0.01 | 0.37 – 0.40 | 0.38 ^***^ | 0.01 | 0.37 – 0.40 | 0.38 ^***^ | 0.01 | 0.37 – 0.40 |
| Asked mediator items this round (M) | 0.08 ^*^ | 0.03 | 0.02 – 0.14 | 0.08 ^*^ | 0.03 | 0.02 – 0.14 | 0.05 | 0.14 | -0.23 – 0.33 |
| H x Alter_give to_ is a bot |  |  |  | -0.73 ^**^ | 0.23 | -1.17 –  -0.29 | -0.75 ^**^ | 0.23 | -1.19 – -0.30 |
| H x Alter_receive from_ is a bot |  |  |  | -0.09 | 0.23 | -0.54 – 0.35 | -0.08 | 0.23 | -0.52 – 0.37 |
| H x M |  |  |  |  |  |  | -0.03 | 0.20 | -0.41 – 0.36 |
| Alter_give to_ is a bot x M |  |  |  |  |  |  | -0.03 | 0.11 | -0.24 – 0.19 |
| Alter_receive from_ is a bot x M |  |  |  |  |  |  | 0.08 | 0.11 | -0.14 – 0.29 |
| H x Alter_give to_ is a bot x M |  |  |  |  |  |  | 0.10 | 0.15 | -0.20 – 0.39 |
| H x Alter_receive from_ is a bot x M |  |  |  |  |  |  | -0.08 | 0.15 | -0.38 – 0.22 |
| Observations | 23416 | | | 23416 | | | 23416 | | |
| Marginal R^2^ / Conditional R^2^ | 0.199 / 0.638 | | | 0.202 / 0.639 | | | 0.202 / 0.639 | | |
| AIC | 101017.673 | | | 101011.858 | | | 101035.008 | | |
| ** p<0.05 ** p<0.01 *** p<0.001* | | | | | | | | | |

**Note:** Multilevel linear models with random intercepts for the multiple decision-rounds nested in participants (N = 2,192) nested in networks (N = 1,293) with one real other participant. Round ranges from 0 to 10. The first round decisions for participants in the A position are dropped because they did not receive information about the amount their alter had given in the first round, by necessity. The outcome variable is the number of tokens ego gave their alter, ranging from 0 to 10.

**Idleness in the *chain reciprocity study***

In the chain reciprocity study, occasionally, one of the participants in the network went “idle” (failed to make a decision about sending their tokens and clicking the button to continue after more than 90 seconds). They were told in advance that if that happened, the computer would make a decision for them for that round so that the real-time study could progress normally for other participants and finish within the allotted time. 79 participants (or 3.7% of our sample) went idle at least one time across the rounds of the study (in 117 rounds total). Of those, 59 went idle only once. Twenty participants went idle more than once (8 went idle twice, 7 went idle three times, 4 went idle four times, and 1 went idle five times). Participants who went idle were eligible to return for subsequent rounds. If they did, they were required to click through to see the decision(s) that the computer had made on their behalf (the computer program engaged in strong reciprocity, i.e., giving the alter the same amount of tokens that the alter had given their own alter) for the round(s) they missed before they could “catch up” and make a decision in the current round.

We probed idleness in the chain reciprocity study to ensure it did not impact the validity of our results in several ways. First, it is possible that idleness was not randomly distributed across conditions. To assess this, we modelled the likelihood of going idle in a given round (versus not going idle). The predictors were those used in our key models for the chain reciprocity study– condition (hybrid versus baseline), whether ego was positioned in the chain such that they could give to a bot, whether ego was positioned to receive from a bot, along with the amount their alter had given and round. Model 1 in Table S6 corresponds to Model 1 in Table 1 of the main text, showing just main effects. None of our predictors were associated with going idle in the round, except *round*– participants were less likely to go idle in later rounds of the study (OR = 0.90, *p* < 0.01). Model 2 in Table S6 corresponds to Model 2 in Table 1 of the main text, containing interactions between the hybrid condition (where participants knew they were interacting with bots) and whether the participant was positioned such that they could give to or receive from a bot. Again, nothing predicted going idle except for *round* (OR = 0.90, *p* < 0.01). This suggests that participants were about equally likely to go idle across conditions and based on other important features of our study (e.g., the amount they learned their alter had given did not impact idleness).

**Table S6. Predicting going idle in the round, *chain reciprocity study***

|  | **Model 1** | | | **Model 2** | | |
| --- | --- | --- | --- | --- | --- | --- |
| *Predictors* | *OR* | *SE* | *95% CI* | *OR* | *SE* | *95% CI* |
| Intercept | 0.00 ^***^ | 0.00 | 0.00 – 0.00 | 0.00 ^***^ | 0.00 | 0.00 – 0.00 |
| Hybrid condition (H) | 1.32 | 0.75 | 0.43 – 4.02 | 1.61 | 2.96 | 0.04 – 58.72 |
| The alter that ego can *give to* is a bot | 0.57 | 0.36 | 0.17 – 1.96 | 0.53 | 0.50 | 0.08 – 3.39 |
| The alter that ego can *receive from* is a bot | 1.13 | 0.86 | 0.25 – 5.05 | 1.41 | 1.79 | 0.12 – 16.88 |
| Round | 0.90 ^**^ | 0.03 | 0.84 – 0.97 | 0.90 ^**^ | 0.03 | 0.84 – 0.97 |
| Amount Alter_give to_ gave their alter this round | 1.01 | 0.05 | 0.92 – 1.11 | 1.01 | 0.05 | 0.92 – 1.11 |
| H x Alter_give to_ is a bot |  |  |  | 1.14 | 1.44 | 0.10 – 13.53 |
| H x Alter_receive from_ is a bot |  |  |  | 0.70 | 1.12 | 0.03 – 15.80 |
| Observations | 23530 | | | 23530 | | |
| Marginal R^2^ /  Conditional R^2^ | 0.003 / 0.943 | | | 0.004 / 0.943 | | |
| AIC | 1067.322 | | | 1071.231 | | |
| ** p<0.05 ** p<0.01 *** p<0.001* | | | | | | |

**Note**: Logistic generalized linear models predicting going idle in the round (versus not going idle in the round), with random intercepts for multiple decision-rounds nested in participants (N = 2,192) nested in networks (N = 1,293) with one real other participant. Round ranges from 0 to 10. The first round decisions for participants in the A position are dropped because they did not receive information about the amount their alter had given in the first round, by necessity.

Next, participants who went idle at some point during the study may have been more distracted or otherwise different from participants that did not go idle. Therefore, we also assessed whether having gone idle at any point in the study predicted giving behavior, and whether controlling for the participant ever going idle impacted our key findings. Model 1 in Table S7 corresponds to Model 1 in Table 1 of the main text. It includes our main effects, but with an additional term for having ever gone idle during the study. Model 2 in Table S7 corresponds to Model 2 in Table 1 of the main text; it includes the interactions between the hybrid condition (where participants knew they were interacting with bots) and whether the participant could give to or receive from a bot. In both models, ever going idle during the study did not predict giving (both ORs = -0.35, *p*s = 0.14 and 0.15 respectively). And the key findings (the significant main effect of the *hybrid condition* in Model 1, and the significant *hybrid condition x the alter ego can give to is a bot* interaction in Model 2) remain unchanged with the inclusion of the term for ever going idle. Model 3 adds an additional term for whether *anyone in the group ever went idle*; again, our key results were not impacted. Thus, we conclude that participants that went idle did not differ in their giving behavior significantly from those that did not, and that our results are not affected by idleness.

**Table S7. Key models, *chain reciprocity study,* controlling for ever going idle**

|  | **Model 1** | | | **Model 2** | | | **Model 3** | | |
| --- | --- | --- | --- | --- | --- | --- | --- | --- | --- |
| *Predictors* | *Est.* | *SE* | *95% CI* | *Est.* | *SE* | *95% CI* | *Est.* | *SE* | *95% CI* |
| Intercept | 4.87 ^***^ | 0.17 | 4.53 – 5.20 | 4.56 ^***^ | 0.23 | 4.11 – 5.00 | 4.56 ^***^ | 0.23 | 4.12 – 5.01 |
| Participant ever went idle | -0.35 | 0.24 | -0.82 – 0.12 | -0.35 | 0.24 | -0.82 – 0.12 | -0.25 | 0.32 | -0.87 – 0.38 |
| Hybrid condition (H) | -0.54 ^***^ | 0.10 | -0.73 – -0.34 | 0.07 | 0.31 | -0.53 – 0.67 | 0.07 | 0.31 | -0.53 – 0.68 |
| The alter that ego can *give to* is a bot | -0.17 | 0.11 | -0.40 – 0.05 | 0.20 | 0.16 | -0.12 – 0.52 | 0.20 | 0.16 | -0.12 – 0.52 |
| The alter that ego can *receive from* is a bot | -0.07 | 0.11 | -0.29 – 0.15 | -0.02 | 0.16 | -0.35 – 0.30 | -0.03 | 0.16 | -0.35 – 0.30 |
| Round | 0.02 ^***^ | 0.00 | 0.01 – 0.02 | 0.02 ^***^ | 0.00 | 0.01 – 0.02 | 0.02 ^***^ | 0.00 | 0.01 – 0.02 |
| Amount Alter_give to_ gave their alter this round | 0.38 ^***^ | 0.01 | 0.37 – 0.39 | 0.38 ^***^ | 0.01 | 0.37 – 0.39 | 0.38 ^***^ | 0.01 | 0.37 – 0.39 |
| H x Alter_give to_ is a bot |  |  |  | -0.73 ^**^ | 0.23 | -1.17 – -0.29 | -0.73 ^**^ | 0.23 | -1.17 – -0.29 |
| H x Alter_receive from_ is a bot |  |  |  | -0.09 | 0.23 | -0.54 – 0.35 | -0.09 | 0.23 | -0.54 – 0.35 |
| Anyone in the group ever went idle |  |  |  |  |  |  | -0.13 | 0.27 | -0.65 – 0.39 |
| Observations | 23416 | | | 23416 | | | 23416 | | |
| Marginal R^2^ / Conditional R^2^ | 0.199 / 0.638 | | | 0.202 / 0.639 | | | 0.202 / 0.639 | | |
| AIC | 101017.909 | | | 101012.155 | | | 101014.734 | | |
| ** p<0.05 ** p<0.01 *** p<0.001* | | | | | | | | | |

**Note:** Multilevel linear models with random intercepts for the multiple decision-rounds nested in participants (N = 2,192) nested in networks (N = 1,293) with one real other participant. Round ranges from 0 to 10. The first round decisions for participants in the A position are dropped because they did not receive information about the amount their alter had given in the first round, by necessity. The outcome variable is the number of tokens ego gave their alter, ranging from 0 to 10.

As a final check, we removed the 79 participants who ever went idle from our data and re-ran our key models. In these models, we again find no substantive differences in our results from those models where participants who ever went idle were included. Model 1 in Table S8 corresponds to Model 1 in Table 1 of the main text, containing the main effects, but it drops the 79 participants who ever went idle during the study. Model 2 corresponds to Model 2 in Table 1 of the main text; it includes the interactions but drops participants who ever went idle. Our key results are unaffected when we include only participants who did not go idle during the study.

**Table S8. Key models, *chain reciprocity study,* participants who ever went idle excluded**

|  | **Model 1** | | | **Model 2** | | |
| --- | --- | --- | --- | --- | --- | --- |
| *Predictors* | *Est.* | *SE* | *95% CI* | *Est.* | *SE* | *95% CI* |
| Intercept | 4.77 ^***^ | 0.17 | 4.44 – 5.11 | 4.42 ^***^ | 0.23 | 3.97 – 4.87 |
| Hybrid condition (H) | -0.55 ^***^ | 0.10 | -0.75 – -0.36 | 0.14 | 0.31 | -0.47 – 0.74 |
| The alter that ego can *give to* is a bot | -0.16 | 0.11 | -0.38 – 0.07 | 0.25 | 0.16 | -0.07 – 0.57 |
| The alter that ego can *receive from* is a bot | -0.03 | 0.11 | -0.25 – 0.20 | 0.04 | 0.16 | -0.28 – 0.36 |
| Round | 0.02 ^***^ | 0.00 | 0.01 – 0.02 | 0.02 ^***^ | 0.00 | 0.01 – 0.02 |
| Amount Alter_give to_ gave their alter this round | 0.39 ^***^ | 0.01 | 0.38 – 0.40 | 0.39 ^***^ | 0.01 | 0.38 – 0.40 |
| H x Alter_give to_ is a bot |  |  |  | -0.80 ^***^ | 0.23 | -1.25 – -0.35 |
| H x Alter_receive from_ is a bot |  |  |  | -0.12 | 0.23 | -0.57 – 0.32 |
| Observations | 22681 | | | 22681 | | |
| Marginal R^2^ /  Conditional R^2^ | 0.208 / 0.640 | | | 0.212 / 0.641 | | |
| AIC | 97643.424 | | | 97635.779 | | |
| ** p<0.05 ** p<0.01 *** p<0.001* | | | | | | |

**Note:** Multilevel linear models with random intercepts for the multiple decision-rounds nested in participants (N = 2,113) nested in networks (N = 1,282) with one real other participant. Round ranges from 0 to 10. The first round decisions for participants in the A position are dropped because they did not receive information about the amount their alter had given in the first round, by necessity. Participants who ever went idle during the study are also dropped. The outcome variable is the number of tokens ego gave their alter, ranging from 0 to 10.

**Robustness**

*Binarizing giving behavior in the* chain reciprocity study

We assessed the robustness of our models in several ways. First, we examined if our effects hold when using different modelling choices. Specifically, for the chain reciprocity study, participants could ostensibly give any number of tokens, from 0 to 10, to their alter. But in the raw data, decisions were heavily clustered at the end- and mid-points of the possible range of choices (i.e., at 0, 5, and 10). This can be seen in Figure S3. Of all giving decisions made in the baseline condition, 59.1% were to give the entire ten-token endowment; in the hybrid condition, it was 47.7%. As a result, we conducted additional (logistic generalized linear) models using the binary outcome of giving the full 10-token endowment versus not giving the full endowment. These models also account for the dependencies in the data with random intercepts for decisions made within participants, and participants nested in networks with a real other participant.


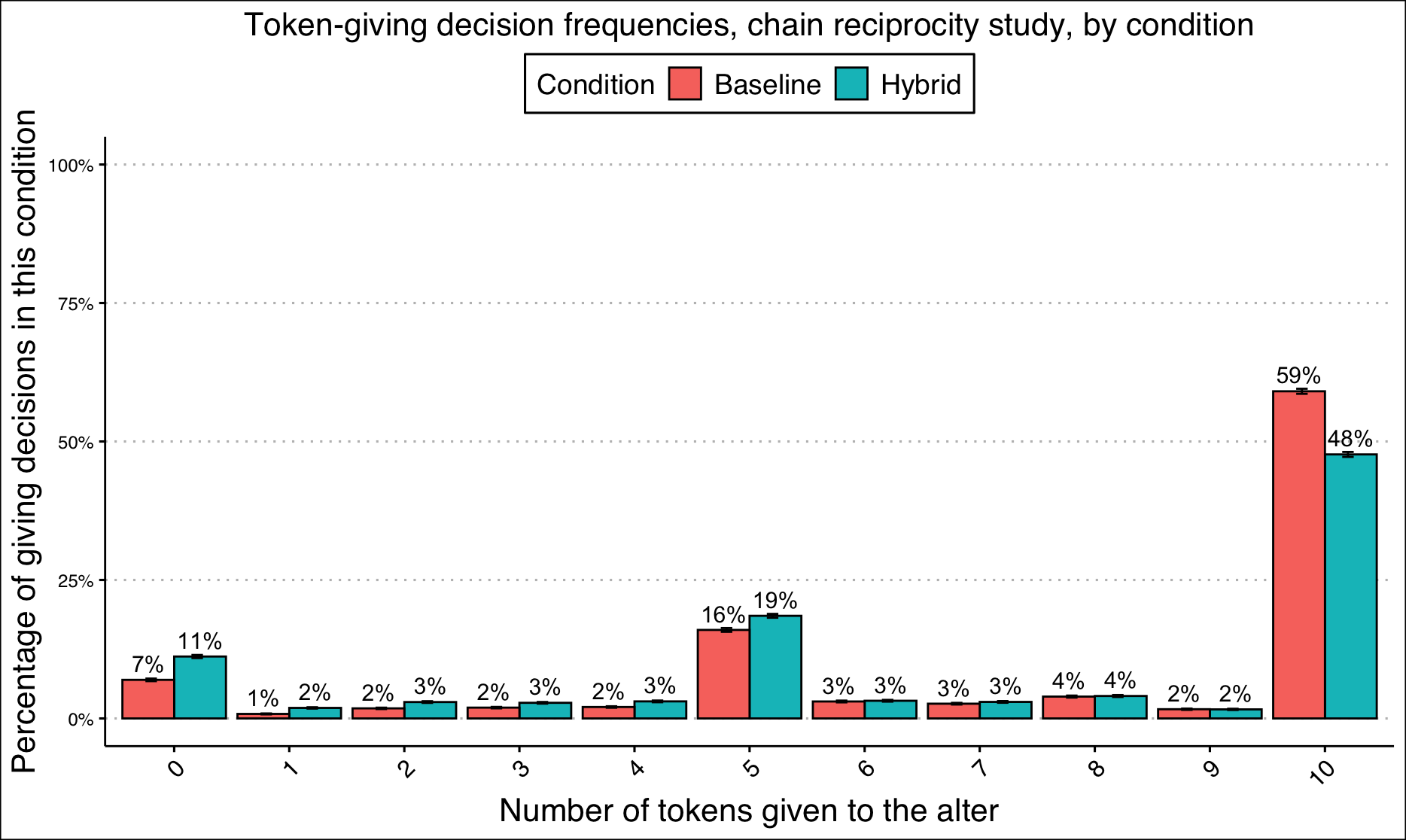


**Figure S3. Token-giving decision frequencies, *chain reciprocity study*, by condition**

**Note:** Error bars represent 95% confidence intervals.

Models 1 and 2 in Table S9 below correspond to Models 1 and 2 in Table 1 of the main text, but with the outcome binarized (the participant gave the entire ten-token endowment versus did not give the entire endowment). Our key findings are very similar to those when the outcome is modelled linearly– participants were less likely to give their entire endowment in the hybrid condition than in the baseline condition (Model 1, OR = 0.30, *p* < 0.001) and this was driven by those who were positioned such that they could provide benefits to a known bot (Model 2, OR = 0.14, *p* < 0.001). There is a difference worth noting, however: our main models discussed in the main text do not find evidence that participants are less generous when they know that they are positioned such that a bot can reward their reputation. But in the binary outcome version in Table S9, participants were less likely to give their entire endowment when they knew that a bot was able to give to them (OR = 0.29, *p* < 0.01). Because this finding is sensitive to model specification (unlike our other findings, which hold across model specifications) and because we pre-registered the modelling strategy we reported in the main text (with giving operationalized on the 0-10 scale), we interpret this latter finding with caution. Our general conclusion is that the results we report in the main text hold to this alternative modelling strategy.

**Table S9. Key models, *chain reciprocity study,* giving the entire endowment versus not giving the entire endowment**

|  | **Model 1** | | | **Model 2** | | |
| --- | --- | --- | --- | --- | --- | --- |
| *Predictors* | *OR* | *SE* | *95% CI* | *OR* | *SE* | *95% CI* |
| Intercept | 0.14 ^***^ | 0.05 | 0.07 – 0.30 | 0.04 ^***^ | 0.02 | 0.02 – 0.11 |
| Hybrid condition (H) | 0.30 ^***^ | 0.07 | 0.20 – 0.46 | 3.24 | 2.16 | 0.87 – 11.98 |
| The alter that ego can *give to* is a bot | 0.85 | 0.20 | 0.54 – 1.36 | 2.41 ^*^ | 0.83 | 1.23 – 4.73 |
| The alter that ego can *receive from* is a bot | 0.98 | 0.23 | 0.61 – 1.56 | 1.95 | 0.68 | 0.99 – 3.86 |
| Round | 1.03 ^***^ | 0.01 | 1.02 – 1.05 | 1.03 ^***^ | 0.01 | 1.02 – 1.05 |
| Amount alter_give to_ gave their alter this round | 1.52 ^***^ | 0.02 | 1.49 – 1.56 | 1.52 ^***^ | 0.02 | 1.48 – 1.56 |
| H x Alter_give to_ is a bot |  |  |  | 0.14 ^***^ | 0.07 | 0.06 – 0.36 |
| H x Alter_receive from_ is a bot |  |  |  | 0.29 ^**^ | 0.14 | 0.11 – 0.73 |
| Observations | 23416 | | | 23416 | | |
| Marginal R^2^ /  Conditional R^2^ | 0.134 / 0.834 | | | 0.144 / 0.832 | | |
| AIC | 15243.392 | | | 15235.408 | | |
| ** p<0.05 ** p<0.01 *** p<0.001* | | | | | | |

**Note:** Logistic generalized linear models with random intercepts for the multiple decision-rounds nested in participants (N = 2,192) nested in networks (N = 1,293) with one real other participant. Round ranges from 0 to 10. The first round decisions for participants in the A position are dropped because they did not receive information about the amount their alter had given in the first round, by necessity. The outcome variable is whether the participant gave their entire endowment to the alter (versus did not give the entire endowment to the alter).

*Multilevel modelling versus clustered standard errors*

Next, while multilevel modelling is one strategy to account for dependencies in our nested data (i.e., rounds nested in participants, nested in a network with one real other participant), another is using clustered standard errors; both techniques accomplish similar tasks [(4–6)](https://www.zotero.org/google-docs/?R4sw7I). To ensure that our results held regardless of which modelling strategy we used, we re-ran our key models for both studies using clustered standard errors instead of multilevel linear models. Across all models, our results held when using clustered standard errors instead of multilevel modelling. Tables S10-S16 below show each model we show in the main text in the Model 1-MLM column, followed by the clustered standard error version in the Model 2-CSE column, for easy comparison.

**Table S10. Multilevel modelling versus clustered standard errors, *chain reciprocity study*: Model 1 in Table 1 of the main text**

|  | **Model 1 - MLM** | | | **Model 2 - CSE** | | |
| --- | --- | --- | --- | --- | --- | --- |
| *Predictors* | *Est.* | *SE* | *95% CI* | *Est.* | *SE* | *95% CI* |
| Intercept | 4.85 ^***^ | 0.17 | 4.52 – 5.18 | 2.68 ^***^ | 0.16 | 2.37 – 2.99 |
| Hybrid condition (H) | -0.54 ^***^ | 0.10 | -0.73 – -0.35 | -0.34 ^***^ | 0.06 | -0.46 – -0.21 |
| The alter that ego can *give to* is a bot | -0.17 | 0.11 | -0.39 – 0.05 | -0.14 | 0.09 | -0.32 – 0.03 |
| The alter that ego can *receive from* is a bot | -0.07 | 0.11 | -0.29 – 0.15 | 0.01 | 0.09 | -0.16 – 0.19 |
| Round | 0.02 ^***^ | 0.00 | 0.01 – 0.02 | 0.02 ^***^ | 0.00 | 0.02 – 0.03 |
| Amount Alter_give to_ gave their alter this round | 0.38 ^***^ | 0.01 | 0.37 – 0.39 | 0.65 ^***^ | 0.01 | 0.62 – 0.68 |
| Observations | 23416 | | | 23416 | | |
| Marginal R^2^ / Conditional R^2^ | 0.199 / 0.638 | | | 0.428 / 0.428 | | |
| AIC | 101017.035 | | | 111146.325 | | |
| ** p<0.05 ** p<0.01 *** p<0.001* | | | | | | |

**Note**: Model 1 is a multilevel linear model with random intercepts for the multiple decision-rounds nested in participants (N = 2,192) nested in networks (N = 1,293) with one real other participant. It corresponds to Model 1 in Table 1 of the main text. Model 2 is an OLS regression model with standard errors clustered at the participant and group levels.

**Table S11. Multilevel modelling versus clustered standard errors, *chain reciprocity study*: Model 2 in Table 1 of the main text**

|  | **Model 1 - MLM** | | | **Model 2 - CSE** | | |
| --- | --- | --- | --- | --- | --- | --- |
| *Predictors* | *Est.* | *SE* | *95% CI* | *Est.* | *SE* | *95% CI* |
| Intercept | 4.54 ^***^ | 0.23 | 4.10 – 4.99 | 2.49 ^***^ | 0.17 | 2.16 – 2.82 |
| Hybrid condition (H) | 0.07 | 0.31 | -0.54 – 0.67 | 0.03 | 0.19 | -0.33 – 0.40 |
| The alter that ego can *give to* is a bot | 0.21 | 0.16 | -0.11 – 0.53 | 0.17 | 0.12 | -0.08 – 0.41 |
| The alter that ego can *receive from* is a bot | -0.03 | 0.16 | -0.35 – 0.30 | -0.04 | 0.12 | -0.28 – 0.20 |
| Round | 0.02 ^***^ | 0.00 | 0.01 – 0.02 | 0.02 ^***^ | 0.00 | 0.02 – 0.03 |
| Amount Alter_give to_ gave their alter this round | 0.38 ^***^ | 0.01 | 0.37 – 0.39 | 0.65 ^***^ | 0.01 | 0.62 – 0.68 |
| H x Alter_give to_ is a bot | -0.73 ^**^ | 0.23 | -1.17 – -0.29 | -0.60 ^***^ | 0.18 | -0.95 – -0.26 |
| H x Alter_receive from_ is a bot | -0.09 | 0.23 | -0.54 – 0.35 | 0.10 | 0.18 | -0.25 – 0.45 |
| Observations | 23416 | | | 23416 | | |
| Marginal R^2^ / Conditional R^2^ | 0.202 / 0.639 | | | 0.430 / 0.430 | | |
| AIC | 101011.223 | | | 111080.735 | | |
| ** p<0.05 ** p<0.01 *** p<0.001* | | | | | | |

**Note**: Model 1 is a multilevel linear model with random intercepts for the multiple decision-rounds nested in participants (N = 2,192) nested in networks (N = 1,293) with one real other participant. It corresponds to Model 2 in Table 1 of the main text. Model 2 is an OLS regression model with standard errors clustered at the participant and group levels.

**Table S12. Multilevel modelling versus clustered standard errors, *one-shot giving study*: Model 1 in Table 2 of the main text for token-giving when reputational giving was and was not possible**

|  | **Model 1 - MLM** | | | **Model 2 - CSE** | | |
| --- | --- | --- | --- | --- | --- | --- |
| *Predictors* | *OR* | *SE* | *95% CI* | *OR* | *SE* | *95% CI* |
| Intercept | 1.20 ^*^ | 0.09 | 1.04 – 1.38 | 1.13 ^**^ | 0.05 | 1.04 – 1.22 |
| Reputational giving is possible | 4.03 ^***^ | 0.19 | 3.66 – 4.43 | 2.11 ^***^ | 0.06 | 1.99 – 2.24 |
| The alter that A can give to (B) is a bot | 0.16 ^***^ | 0.01 | 0.15 – 0.18 | 0.38 ^***^ | 0.01 | 0.35 – 0.41 |
| The alter that ego can receive from (C) is a bot | 0.99 | 0.05 | 0.91 – 1.09 | 1.00 | 0.02 | 0.96 – 1.04 |
| Observations | 15880 | | | 15880 | | |
| Marginal R^2^ / Conditional R^2^ | 0.118 / 0.702 | | | 0.062 / 0.062 | | |
| AIC | 15973.692 | | | 20656.800 | | |
| ** p<0.05 ** p<0.01 *** p<0.001* | | | | | | |

**Note**: Model 1 is a multilevel logistic model with random intercepts for multiple decision-rounds nested within participants (N = 1,985). It corresponds to Model 1 in Table 2 of the main text. Model 2 is a logistic regression model with standard errors clustered at the participant level.

**Table S13. Multilevel modelling versus clustered standard errors, *one-shot giving study*: Model 2 in Table 2 of the main text for token-giving when reputational giving was and was not possible**

|  | **Model 1 - MLM** | | | **Model 2 - CSE** | | |
| --- | --- | --- | --- | --- | --- | --- |
| *Predictors* | *OR* | *SE* | *95% CI* | *OR* | *SE* | *95% CI* |
| Intercept | 1.35 ^***^ | 0.11 | 1.15 – 1.58 | 1.20 ^***^ | 0.05 | 1.11 – 1.31 |
| Reputational giving is possible (RG) | 3.14 ^***^ | 0.25 | 2.68 – 3.67 | 1.83 ^***^ | 0.07 | 1.69 – 1.98 |
| The alter that A can give to (B) is a bot | 0.13 ^***^ | 0.01 | 0.11 – 0.15 | 0.33 ^***^ | 0.02 | 0.30 – 0.36 |
| The alter that ego can receive from (C) is a bot | 0.99 | 0.06 | 0.87 – 1.13 | 1.00 | 0.03 | 0.94 – 1.05 |
| RG x B is a bot | 1.63 ^***^ | 0.15 | 1.36 – 1.95 | 1.33 ^***^ | 0.06 | 1.22 – 1.45 |
| RG x C is a bot | 1.01 | 0.09 | 0.84 – 1.20 | 1.00 | 0.04 | 0.93 – 1.09 |
| Observations | 15880 | | | 15880 | | |
| Marginal R^2^ /  Conditional R^2^ | 0.119 / 0.703 | | | 0.063 / 0.062 | | |
| AIC | 15949.144 | | | 20642.500 | | |
| ** p<0.05 ** p<0.01 *** p<0.001* | | | | | | |

**Note**: Model 1 is a multilevel logistic model with random intercepts for multiple decision-rounds nested within participants (N = 1,985). It corresponds to Model 2 in Table 2 of the main text. Model 2 is a logistic regression model with standard errors clustered at the participant level.

**Table S14. Multilevel modelling versus clustered standard errors, *one-shot giving study*: Model 3 in Table 2 of the main text for token-giving when reputational giving was and was not possible**

|  | **Model 1 - MLM** | | | **Model 2 - CSE** | | |
| --- | --- | --- | --- | --- | --- | --- |
| *Predictors* | *OR* | *SE* | *95% CI* | *OR* | *SE* | *95% CI* |
| Intercept | 1.42 ^***^ | 0.12 | 1.20 – 1.68 | 1.24 ^***^ | 0.06 | 1.13 – 1.35 |
| Reputational giving is possible (RG) | 3.35 ^***^ | 0.31 | 2.79 – 4.02 | 1.89 ^***^ | 0.08 | 1.73 – 2.06 |
| The alter that A can give to (B) is a bot | 0.11 ^***^ | 0.01 | 0.10 – 0.14 | 0.31 ^***^ | 0.02 | 0.28 – 0.35 |
| The alter that ego can receive from (C) is a bot | 0.90 | 0.08 | 0.76 – 1.07 | 0.94 | 0.04 | 0.88 – 1.02 |
| RG x B is a bot | 1.48 ^**^ | 0.19 | 1.15 – 1.91 | 1.26 ^***^ | 0.08 | 1.12 – 1.42 |
| RG x C is a bot | 0.89 | 0.11 | 0.69 – 1.14 | 0.94 | 0.05 | 0.85 – 1.05 |
| Bot is a bot x C is a bot | 1.24 | 0.16 | 0.96 – 1.60 | 1.12 | 0.07 | 1.00 – 1.26 |
| RG x Bot is a bot x C is a bot | 1.21 | 0.22 | 0.85 – 1.74 | 1.10 | 0.09 | 0.94 – 1.29 |
| Observations | 15880 | | | 15880 | | |
| Marginal R^2^ / Conditional R^2^ | 0.120 / 0.704 | | | 0.063 / 0.063 | | |
| AIC | 15940.216 | | | 20639.645 | | |
| ** p<0.05 ** p<0.01 *** p<0.001* | | | | | | |

**Note**: Model 1 is a multilevel logistic model with random intercepts for multiple decision-rounds nested within participants (N = 1,985). It corresponds to Model 3 in Table 2 of the main text. Model 2 is a logistic regression model with standard errors clustered at the participant level.

**Table S15. Multilevel modelling versus clustered standard errors, *one-shot giving study*: Model 1 in Table 3 of the main text for token-giving when rewarding reputations**

|  | **Model 1 - MLM** | | | **Model 2 - CSE** | | |
| --- | --- | --- | --- | --- | --- | --- |
| *Predictors* | *OR* | *SE* | *95% CI* | *OR* | *SE* | *95% CI* |
| Intercept | 0.10 ^***^ | 0.01 | 0.09 – 0.12 | 0.25 ^***^ | 0.01 | 0.23 – 0.28 |
| The alter that ego can give to (A) is a bot | 0.25 ^***^ | 0.01 | 0.22 – 0.27 | 0.44 ^***^ | 0.02 | 0.40 – 0.47 |
| The alter that A can give to (B) is a bot | 0.99 | 0.05 | 0.90 – 1.09 | 0.99 | 0.02 | 0.95 – 1.04 |
| A gave their token to B | 25.66 ^***^ | 1.64 | 22.65 – 29.08 | 7.11 ^***^ | 0.38 | 6.40 – 7.91 |
| Observations | 15880 | | | 15880 | | |
| Marginal R^2^ / Conditional R^2^ | 0.271 / 0.714 | | | 0.161 / 0.161 | | |
| AIC | 14153.310 | | | 17193.785 | | |
| ** p<0.05 ** p<0.01 *** p<0.001* | | | | | | |

**Note**: Model 1 is a multilevel logistic model with random intercepts for multiple decision-rounds nested within participants (N = 1,985). It corresponds to Model 1 in Table 3 of the main text. Model 2 is a logistic regression model with standard errors clustered at the participant level.

**Table S16. Multilevel modelling versus clustered standard errors, *one-shot giving study*: Model 2 in Table 3 of the main text for token-giving when rewarding reputations**

|  | **Model 1 - MLM** | | | **Model 2 - CSE** | | |
| --- | --- | --- | --- | --- | --- | --- |
| *Predictors* | *OR* | *SE* | *95% CI* | *OR* | *SE* | *95% CI* |
| Intercept | 0.06 ^***^ | 0.01 | 0.05 – 0.07 | 0.19 ^***^ | 0.01 | 0.17 – 0.22 |
| The alter that ego can give to (A) is a bot | 0.58 ^***^ | 0.07 | 0.46 – 0.73 | 0.70 ^***^ | 0.05 | 0.61 – 0.80 |
| The alter that A can give to (B) is a bot | 1.73 ^***^ | 0.18 | 1.42 – 2.12 | 1.43 ^***^ | 0.08 | 1.27 – 1.60 |
| A gave their token to B | 61.12 ^***^ | 6.89 | 49.00 – 76.24 | 11.09 ^***^ | 0.79 | 9.65 – 12.74 |
| A is a bot x B is a bot | 0.50 ^***^ | 0.08 | 0.36 – 0.68 | 0.63 ^***^ | 0.05 | 0.54 – 0.75 |
| A is a bot x A gave their token to B | 0.26 ^***^ | 0.04 | 0.19 – 0.34 | 0.50 ^***^ | 0.04 | 0.43 – 0.58 |
| B is a bot x A gave their token to B | 0.39 ^***^ | 0.05 | 0.30 – 0.51 | 0.57 ^***^ | 0.04 | 0.50 – 0.65 |
| A is a bot x B is a bot x A gave their token to B | 2.96 ^***^ | 0.60 | 1.99 – 4.40 | 1.93 ^***^ | 0.19 | 1.59 – 2.34 |
| Observations | 15880 | | | 15880 | | |
| Marginal R^2^ / Conditional R^2^ | 0.265 / 0.720 | | | 0.163 / 0.163 | | |
| AIC | 14052.677 | | | 17152.351 | | |
| ** p<0.05 ** p<0.01 *** p<0.001* | | | | | | |

**Note**: Model 1 is a multilevel logistic model with random intercepts for multiple decision-rounds nested within participants (N = 1,985). It corresponds to Model 2 in Table 3 of the main text. Model 2 is a logistic regression model with standard errors clustered at the participant level.

*Bootstrapping*

Finally, we conducted bootstrapping on our key models for both studies to evaluate the robustness of our results and the stability of our models. Across all analyses, our main findings hold in the bootstrapped analyses, which indicates that our results are not due to overfitting or outliers. Tables S17-S23 below show the odds ratios, SEs, and 95% CIs from our key models, followed by the bootstrapped results: bootstrapped estimates or odds ratios, SEs, and 95% CIs (N = 1000 simulations, percentile method).

**Table S17. Bootstrapped results from the *chain reciprocity study*: Model 1 in Table 1 in the main text**

| *Term* | *Model*  *Est* | *Model*  *SE* | *Model*  *95% CI* | *Boot*  *Mean Est* | *Boot*  *SE* | *BootEst*  *95% CI* |
| --- | --- | --- | --- | --- | --- | --- |
| Intercept | 4.85 | 0.17 | 4.52 - 5.18 | 4.85 | 0.17 | 4.51 - 5.20 |
| Hybrid condition | -0.54 | 0.10 | -0.73 - -0.35 | -0.54 | 0.10 | -0.74 - -0.33 |
| The alter that ego can *give to* is a bot | -0.17 | 0.11 | -0.39 - 0.05 | -0.17 | 0.12 | -0.39 - 0.06 |
| The alter that ego can *receive from* is a bot | -0.07 | 0.11 | -0.29 - 0.15 | -0.07 | 0.11 | -0.29 - 0.15 |
| Round | 0.02 | 0.00 | 0.01 - 0.02 | 0.02 | 0.00 | 0.01 - 0.02 |
| Amount Alter_give to_ gave their alter this round | 0.38 | 0.01 | 0.37 - 0.39 | 0.38 | 0.01 | 0.37 - 0.39 |

**Table S18. Bootstrapped results from the *chain reciprocity study*: Model 2 in Table 1 of the main text**

| *Term* | *Model Est* | *Model*  *SE* | *Model*  *95% CI* | *Boot*  *Mean Est* | *Boot*  *SE* | *BootEst*  *95% CI* |
| --- | --- | --- | --- | --- | --- | --- |
| Intercept | 4.54 | 0.23 | 4.1 - 4.99 | 4.55 | 0.22 | 4.11 - 5.00 |
| Hybrid condition (H) | 0.07 | 0.31 | -0.54 - 0.67 | 0.06 | 0.31 | -0.60 - 0.64 |
| The alter that ego can *give to* is a bot | 0.21 | 0.16 | -0.11 - 0.53 | 0.21 | 0.16 | -0.12 - 0.51 |
| The alter that ego can *receive from* is a bot | -0.03 | 0.16 | -0.35 - 0.3 | -0.03 | 0.16 | -0.33 - 0.30 |
| Round | 0.02 | 0.00 | 0.01 - 0.02 | 0.02 | 0.00 | 0.01 - 0.02 |
| Amount Alter_give to_ gave their alter this round | 0.38 | 0.01 | 0.37 - 0.39 | 0.38 | 0.01 | 0.37 - 0.39 |
| H x Alter_give to_ is a bot | -0.73 | 0.23 | -1.17 - -0.29 | -0.73 | 0.23 | -1.17 - -0.26 |
| H x Alter_receive from_ is a bot | -0.09 | 0.23 | -0.54 - 0.35 | -0.09 | 0.23 | -0.52 - 0.35 |

**Table S19. Bootstrapped results from the *one-shot giving study:* Model 1 in Table 2 of the main text for token-giving when reputational giving was and was not possible**

| *Term* | *Model*  *OR* | *Model*  *SE* | *Model*  *95% CI* | *Boot*  *Mean OR* | *Boot*  *SE* | *Boot*  *95% CI* |
| --- | --- | --- | --- | --- | --- | --- |
| Intercept | 1.20 | 0.09 | 0.15 - 0.18 | 1.20 | 0.09 | 1.04 - 1.37 |
| Reputational giving is possible | 4.03 | 0.19 | 0.91 - 1.09 | 4.01 | 0.20 | 3.66 - 4.44 |
| The alter that ego can give to (B) is a bot | 0.16 | 0.01 | 0.15 - 0.18 | 0.17 | 0.01 | 0.15 - 0.18 |
| The alter that ego can receive from (C) is a bot | 0.99 | 0.05 | 0.91 - 1.09 | 0.99 | 0.05 | 0.91 - 1.09 |

**Table S20. Bootstrapped results from the *one-shot giving study*: Model 2 in Table 2 of the main text for token-giving when reputational giving was and was not possible**

| *Term* | *Model*  *OR* | *Model*  *SE* | *Model*  *95% CI* | *Boot*  *Mean OR* | *Boot*  *SE* | *Boot*  *95% CI* |
| --- | --- | --- | --- | --- | --- | --- |
| Intercept | 1.35 | 0.11 | 1.15 - 1.58 | 1.35 | 0.11 | 1.16 - 1.56 |
| Reputational giving is possible (RG) | 3.14 | 0.25 | 2.68 - 3.67 | 3.14 | 0.25 | 2.69 - 3.64 |
| The alter that ego can give to (B) is a bot | 0.13 | 0.01 | 0.11 - 0.15 | 0.13 | 0.01 | 0.11 - 0.15 |
| The alter that ego can receive from (C) is a bot | 0.99 | 0.06 | 0.87 - 1.13 | 0.99 | 0.06 | 0.88 - 1.12 |
| RG x B is a bot | 1.63 | 0.15 | 1.36 - 1.95 | 1.62 | 0.15 | 1.35 - 1.92 |
| RG x C is a bot | 1.01 | 0.09 | 0.84 - 1.20 | 1.01 | 0.09 | 0.86 - 1.20 |

**Table S21. Bootstrapped results from the *one-shot giving study*: Model 3 in Table 2 of the main text for token-giving when reputational giving was and was not possible**

| *Term* | *Model*  *OR* | *Model*  *SE* | *Model*  *95% CI* | *Boot*  *Mean OR* | *Boot*  *SE* | *Boot*  *95% CI* |
| --- | --- | --- | --- | --- | --- | --- |
| Intercept | 1.42 | 0.12 | 1.2 - 1.68 | 1.42 | 0.12 | 1.21 - 1.66 |
| Reputational giving is possible (RG) | 3.35 | 0.31 | 2.79 - 4.02 | 3.35 | 0.31 | 2.78 - 3.96 |
| The alter that ego can give to (B) is a bot | 0.11 | 0.01 | 0.10 - 0.14 | 0.12 | 0.01 | 0.09 - 0.14 |
| The alter that ego can receive from (C) is a bot | 0.90 | 0.08 | 0.76 - 1.07 | 0.90 | 0.08 | 0.76 - 1.06 |
| RG x B is a bot | 1.48 | 0.19 | 1.15 - 1.91 | 1.47 | 0.19 | 1.14 - 1.89 |
| RG x C is a bot | 0.89 | 0.11 | 0.69 - 1.14 | 0.89 | 0.11 | 0.69 - 1.13 |
| B is a bot x C is a bot | 1.24 | 0.16 | 0.96 - 1.60 | 1.24 | 0.17 | 0.96 - 1.61 |
| RG x B is a bot x C is a bot | 1.21 | 0.22 | 0.85 - 1.74 | 1.22 | 0.22 | 0.84 - 1.73 |

**Table S22. Bootstrapped results from the *one-shot giving study*: Model 1 in Table 3 of the main text for token-giving when rewarding reputations**

| *Term* | *Model*  *OR* | *Model*  *SE* | *Model*  *95% CI* | *Boot*  *Mean OR* | *Boot*  *SE* | *Boot*  *95% CI* |
| --- | --- | --- | --- | --- | --- | --- |
| Intercept | 0.10 | 0.01 | 0.09 - 0.12 | 0.10 | 0.01 | 0.09 - 0.12 |
| The alter that ego can give to (A) is a bot | 0.25 | 0.01 | 0.22 - 0.27 | 0.25 | 0.01 | 0.22 - 0.27 |
| The alter that A could give to (B) is a bot | 0.99 | 0.05 | 0.90 - 1.09 | 0.99 | 0.05 | 0.90 - 1.08 |
| A gave their token to B | 25.66 | 1.64 | 22.65 - 29.08 | 25.29 | 1.69 | 22.44 - 29.15 |

**Table S23. Bootstrapped results from the *one-shot giving study*: Model 2 in Table 3 of the main text for token-giving when rewarding reputations**

| *Term* | *Model*  *OR* | *Model*  *SE* | *Model*  *95% CI* | *Boot*  *Mean OR* | *Boot*  *SE* | *Boot*  *95% CI* |
| --- | --- | --- | --- | --- | --- | --- |
| Intercept | 0.06 | 0.01 | 0.05 - 0.07 | 0.06 | 0.01 | 0.05 - 0.07 |
| The alter that ego can give to (A) is a bot | 0.58 | 0.07 | 0.46 - 0.73 | 0.58 | 0.07 | 0.46 - 0.73 |
| The alter that A could give to (B) is a bot | 1.73 | 0.18 | 1.42 - 2.12 | 1.72 | 0.19 | 1.39 - 2.13 |
| A gave their token to B | 61.12 | 6.89 | 49 - 76.24 | 60.05 | 7.15 | 47.76 - 76.03 |
| A is a bot x B is a bot | 0.50 | 0.08 | 0.36 - 0.68 | 0.50 | 0.08 | 0.36 - 0.67 |
| A is a bot x A gave their token to B | 0.26 | 0.04 | 0.19 - 0.34 | 0.26 | 0.04 | 0.19 - 0.34 |
| B is a bot x A gave their token to B | 0.39 | 0.05 | 0.3 - 0.51 | 0.39 | 0.05 | 0.30 - 0.52 |
| A is a bot x B is a bot x A gave their token to B | 2.96 | 0.60 | 1.99 - 4.4 | 2.93 | 0.62 | 1.98 - 4.44 |

**Supplementary references**

[1. J. P. Heisig, M. Schaeffer, Why You Should Always Include a Random Slope for the Lower-Level Variable Involved in a Cross-Level Interaction. *Eur. Sociol. Rev.* **35**, 258–279 (2019).](https://www.zotero.org/google-docs/?Vf2Ukg)

[2. M. S. Fritz, H. F. Lester, “Mediator Variables” in *Oxford Research Encyclopedia of Psychology*, (2016).](https://www.zotero.org/google-docs/?Vf2Ukg)

[3. A. D. Wu, B. D. Zumbo, Understanding and Using Mediators and Moderators. *Soc. Indic. Res.* **87**, 367–392 (2008).](https://www.zotero.org/google-docs/?Vf2Ukg)

[4. B. C. Cheah, “Clustering standard errors or modeling multilevel data” in *Clustering Standard Errors or Modeling Multilevel Data*, (2009), pp. 2–4.](https://www.zotero.org/google-docs/?Vf2Ukg)

[5. D. McNeish, A practical guide to selecting and blending approaches for clustered data: Clustered errors, multilevel models, and fixed-effect models. *Psychol. Methods* (2023). https://doi.org/10.1037/met0000620.](https://www.zotero.org/google-docs/?Vf2Ukg)

[6. A. Oshchepkov, A. Shirokanova, Bridging the gap between multilevel modeling and economic methods. *Soc. Sci. Res.* **104**, 102689 (2022).](https://www.zotero.org/google-docs/?Vf2Ukg)

1. We intended to collect data from 12 rounds and mediation items after rounds 4, 8, and 12. But due to a programming error, we have valid data for 11 rounds (and valid reputational concern and deserving help items from two rounds, round 4 and round 8). [↑](#footnote-ref-1)
2. This item was not included in the final scale because it could plausibly be negatively correlated with the other two items (specifically, for those alters who gave low amounts or nothing in the round), and dropping it improved Cronbach’s alpha accordingly. [↑](#footnote-ref-2)
